# The effects of a structured communication tool in patients with medically unexplained physical symptoms: a cluster randomized trial

**DOI:** 10.1016/j.eclinm.2023.102262

**Published:** 2023-10-06

**Authors:** Cathrine Abrahamsen, Silje Endresen Reme, Knut Reidar Wangen, Morten Lindbæk, Erik Lønnmark Werner

**Affiliations:** a Faculty of Medicine, Department of General Practice, University of Oslo, Oslo, Norway; b Faculty of Social Sciences, Department of Psychology, University of Oslo, Norway; c Faculty of Medicine, Department of Health Management and Health Economics, University of Oslo, Oslo, Norway

**Keywords:** Medically unexplained symptoms, MUPS, Somatic symptoms, Somatoform disorders, Conversation tools, General practitioners, General physicians, Primary care, Primary health care, Primary medical care, Sick leave, Medical leave

## Abstract

**Background:**

Medically Unexplained Physical Symptoms (MUPS) are prevalent among primary care patients and frequently lead to diminished quality of life, increased healthcare costs, and decreased work participation. We aimed to examine the effects of a work-focused structured communication tool based on cognitive-behavioral therapy in patients with MUPS.

**Methods:**

In a Norwegian two-arm cluster randomized trial, the effectiveness of the structured communication tool Individual Challenge Inventory Tool (ICIT) was compared to usual care for patients with MUPS using a two-arm cluster randomized design. Enrollment period was between March 7 and April 1, 2022. Ten groups (clusters) of 103 General Practitioners (GPs) were randomized to provide the ICIT or usual care for 11 weeks. Patients received two or more sessions with their GP, and outcomes were assessed individually. Primary outcome was patient-reported change in function, symptoms, and quality of life measured by the Patient Global Impression of Change (PGIC). Secondary outcomes included sick leave, work-related self-efficacy (RTW-SE), health-related quality of life (RAND-36), and patient experiences with consultants (PEQ). The trial was registered on ClinicalTrials.gov (NCT05128019).

**Findings:**

A total of 541 patients with MUPS were enrolled in the study. In the intervention group 76% (n = 223) showed a significant overall improvement in function, symptoms, and quality of life as measured by the PGIC, compared to 38% (n = 236) in the usual care group (mean difference −0.8 ([95% CI −1.0 to −0.6]; p < 0.0001). At 11 weeks, the intervention group had a 27-percentage point decrease in sick leave (from 52.0 to 25.2), compared to 4-percentage point decrease (from 49.7 to 45.7) in the usual care group. Furthermore, compared to usual care, the intervention group reported significant improvements in work-related self-efficacy, health-related quality of life, and greater satisfaction with the communication during the consultations. No adverse events were reported.

**Interpretation:**

The implementation of the structured communication tool ICIT in primary care significantly improved patient outcomes and reduced sick leave among patients with MUPS.

**Funding:**

The study was funded by The Norwegian Research Fund for General Practice.

**Research in context**

**Evidence before this study**

We searched for work-oriented cognitive therapy interventions for MUPS patients in primary care up to May 9th, 2023 (Supplementary Material S5). To the best of our knowledge there is no literature on the use of work-focused communication tools for patients with MUPS patients or their impact on sick leave assessments.

**Added value of this study**

This study compromises of 541 patients with MUPS and 103 GPs. The intervention was a work-focused cognitive-behavioral therapy communication tool, named Individual Challenge Inventory Tool (ICIT). No previous GP-led intervention with a structured communication tool has shown similar effects on function, symptoms, sick leave, and quality of life in MUPS patients.

**Implications of all the available evidence**

The GPs use of the structured communication tool ICIT is low-cost and feasible and could positively impact sick leave rates, symptoms, and function in patients with MUPS. It could reduce unnecessary referrals to secondary care and their associated costs. Follow-up studies are needed to replicate and investigate long-term effects.

## Introduction

Medically Unexplained Physical Symptoms (MUPS) are characterized by persistent bodily symptoms and functional impairment that lack an explanation through known medical condition or pathology.

This condition often leads to frustration for both patients and physicians, with patients experiencing dissatisfaction with their medical treatment, feelings of stigmatization, and a sense of not being taken seriously.^1^ Given the necessity for long-term follow-up care, the General practitioner (GP) plays a crucial role in managing patients with MUPS. It is estimated that up to 40% of all consultations in primary care involve patients presenting with MUPS.^2^^,^^3^ Additionally, due to the high number of referrals and further examinations, these patients are also commonly encountered in specialist healthcare.^2^

Patients with MUPS frequently experience psychological distress, social isolation, and a decline in their overall quality of life, resulting in high healthcare utilization and costs associated with sick leave.^3^, ^4^, ^5^ Although cognitive-behavioral therapy (CBT) has demonstrated effectiveness in specialized settings, its efficacy in primary care has been relatively limited.^1^^,^^3^^,^^6^ Moreover, the feasibility of implementing CBT in primary care may be constrained by shorter appointment durations and heavy workloads.^7^ Therefore, we propose the adoption of a communication tool that integrates a practical framework grounded in CBT principles but specifically tailored to the primary care context.

GPs often face challenges when assessing sick leave in patients without a defined diagnosis.^8^ Previous research in primary care has failed to identify interventions that effectively reduce sick leave among patients with MUPS.^9^ In fact, even CBT interventions targeting similar patient groups have encountered difficulties in demonstrating effects om return to work.^10^ Recent studies highlight the importance of explicitly integrating a focus on return to work within interventions to impact sick leave outcomes.^11^ We propose that his approach is also applicable to the treatment of patients with MUPS in primary care. In this context, the communication tool developed by the first author (CA) of this study applies a work-focused CBT approach specifically tailored to the management of patients with MUPS in primary care. We have previously reported on the feasibility and acceptability of this tool.^12^ The aim of the current study is to investigate the effectiveness of this communication tool on:

• Patient-reported outcome of change in function, symptoms, and quality of life.
• Return to work/reduced sick leave.
• Work related self-efficacy.
• Health-related quality of life.
• Patient experiences with the intervention.

## Methods

### Study design

The study employed a two-arm pragmatic cluster randomized trial in Norway, where groups of GPs (clusters) were randomly assigned to either utilize a structured communication tool or provide treatment as usual for patients with MUPS. The use of a cluster randomization design was chosen to ensure the fidelity of the intervention and to prevent GPs from switching between treatments. To ensure proper reporting of the study results, the Consolidated Standards of Reporting Trials (CONSORT) reporting guidelines for cluster randomized trials were followed.^13^

### Participants

Participants in the study were selected by their GP if they were aged 18 years or older and had experienced one or more of 23 physical complaints listed in the "Robbins list" for at least three months^14^ (Table 1). This selection procedure aligns with previous research.^3^ Additionally, participants had to meet the following criteria: (1) functional impairment with sick leave constantly or on and off over a long period of time, and/or (2) withdrawal or avoidance from social activities due to MUPS, (3) gone through adequate medical assessment with no explanatory pathology, and (4) symptom duration of at least three consecutive months to exclude transient ailments.^15^ Participants were excluded from the study if: (1) they did not speak Norwegian sufficiently to answer the questionnaire, (2) they were currently undergoing medical assessment with indications of specific pathology, (3) they did not share the GPs opinion of their complaints and requested further medical examination, or (4) had alcohol or drug addiction.

**Table 1:** Symptoms from the “Robbins List”.

1. Back pain
2. Joint pain
3. Extremity pain
4. Headaches
5. Weakness
6. Fatigue
7. Sleep disturbance
8. Difficulty concentrating
9. Loss of appetite
10. Weight change
11. Restlessness
12. Thoughts slower
13. Chest pain
14. Shortness of breath
15. Palpitations
16. Dizziness
17. Lump in throat
18. Numbness
19. Nausea
20. Loose bowels
21. Gas or bloating
22. Constipation
23. Abdominal pain

All the participants in the study met the same inclusion/exclusion criteria and provided informed consent prior to their participation in the study. To keep the study as close to current practice as possible, patients were selected from the standard patient booking system. No patients were invited to see the GP for the purpose of the study. GPs were instructed to enroll the first ten eligible and willing patients who made appointments in the first four weeks of the study. Participants gave informed consent and completed a pre-consultation questionnaire before their first appointment. The intervention period was from March 4th to May 21st, 2022.

### Randomization and masking

In Norway, a total of 129 GPs enrolled in an open enrollment course that was promoted through the Norwegian Medical Association's class program and a Facebook group for Norwegian GPs. Out of the 129 GPs who enrolled in the course, a total of 103 GPs from various locations throughout Norway met the eligibility criteria of practicing in primary care and willingly provided written informed consent prior to the start of the study. GPs either joined the course individually as the sole representatives from their clinics or participated as a group from the same clinic. To maintain the study's integrity and prevent any cross-contamination, GPs from the same clinic were assigned to the same cluster. For those GPs who joined individually, they were randomly distributed among ten clusters to achieve a balanced distribution of GPs across each cluster, which served as the units of randomization.

The randomization process was conducted in the following manner:

• The 103 General Practitioners (GPs) were divided into ten clusters based on the previously mentioned grouping prior to randomization.
• The names of the GPs within each cluster were securely sealed inside envelopes to ensure confidentiality and integrity of the process.
• To ensure impartiality, an independent staff member from the University of Oslo, who had no affiliation with the research team, was responsible for the selection of envelopes. This staff member alternately chose envelopes to assign the clusters of GPs to either the usual care or intervention group.

It is important to note that although the randomization process determined the allocation of the ten clusters to either the intervention or usual care, each individual GP represented a cluster within the study. Furthermore, eligible participants with MUPS were enrolled in the study by their respective GPs.

Participants were unblinded due to intervention nature, while the study statistician, who also assessed outcomes, was blinded. GPs were blinded to outcome assessments, and participants were instructed not to disclose questionnaire items to their GP as per consent form.

### The intervention

The first author (CA), a practicing GP, developed the communication tool utilized in this study due to the lack of effective tools available to GPs in clinical practice. The tool, known as the "Individual Challenge Inventory Tool (ICIT)," is derived from validated CBT techniques, including problem-solving, behavioral activation, Socratic dialogue, and cognitive restructuring,^16^ the latter especially applied when addressing sick leave. The implementation of ICIT involves the integration of these CBT techniques to develop a detailed activity plan while employing Socratic dialogue to emphasize possibilities rather than limitations (See Supplementary Material S1 for a full description of scientific background of the ICIT). Despite being a transdiagnostic communication tool, ICIT is specifically tailored to assist GPs in efficiently managing patients with MUPS. Its foremost aim is to empower patients, helping them enhance their coping skills in both their daily lives and work settings. The GPs strive to achieve these objectives by utilizing the tool ICIT, following a series of steps: (1) validating patients' feelings, (2) presenting an explanatory model of MUPS, deliberately created to explicate the concept of allostatic overload,^17^ to establishes a mutual understanding of the patients’ complaints, and (3) jointly formulating a written activity plan, such as a "job list," "problem list," or "list of opportunity," depending on the patient’s specific issue. The ICIT's condensed version (Supplementary Material S1) were made available to physicians in a laminated manual, allowing for easy access during patient consultations. Below are three examples demonstrating the application of ICIT based on the patient's problem:

• Overcoming problem overwhelm: The ICIT instructs the GP to create a concise "problem list" in the patient's medical record. Collaboratively, the patient and GP prioritize the problems, distinguishing those that can be addressed immediately from those beyond the patient's control. Subsequently, the patient selects one problem to focus on, and with the help of the GP a suggested solution is registered as an activity plan in the patient's medical record.
• Addressing low energy and reduced self-confidence: The ICIT recommends the use of the "list of opportunity." Within this framework, GPs utilize a predetermined set of Socratic questions to explore how the patient's ailments impact their daily life. The aim is to collaboratively create an activity plan in the patient's medical record, focusing on feasible goals as perceived by the patient. For instance, one question may involve assisting the patient in planning and organizing periods of rest to regain sufficient energy, enabling them to participate in social activities they had previously withdrawn from due to their symptoms.
• When the topic of work participation and sick leave arises, GPs generate a "job list" using four targeted Socratic questions, for example: “What aspects of work would be helpful for you given your current situation?” The participant’s' responses are documented in their medical records as a “job list”. This "job list" can be shared with supervisors for workplace support instead of sick leave. If sick leave is needed, the list is included in the note to guide necessary workplace adjustments. This approach provides a comprehensive assessment of the patient's work abilities to the Norwegian Labor and Welfare Agency (NAV), focusing on capabilities rather than limitations.

To ensure the appropriate and intended use of the ICIT, the GPs were given clear instructions to produce two hard copies of the medical consultation record from each session using the ICIT. One copy for the participant, and the other copy was anonymized and submitted to the research team. As in all CBT treatments, homework assignments are essential. Thus, GPs were mandated to provide a minimum of one follow-up session to review the activity plan. Since participants had the autonomy to schedule their own appointments, we did not set a fixed number of sessions, except for the initial two sessions to address homework follow-up. Participants assigned to the usual care group received their regular treatment from their GP without any specific guidance. The GPs were instructed to treat these patients as they typically would, including the number of consultations required for their specific needs.

### Training of the GP

The training adhered to Bandura's social learning theory, involving attention, theoretical training, role-playing, and video presentations to motivate observation and imitation of generating a "job list", "problem list", or "list of opportunity".^18^ GPs received a 15-h training program comprising of a two-day course with lectures followed by 2 digital meetings after 4 and 8 weeks to guide the GPs to use the structured communication tool ICIT.

### Data collection

A questionnaire was administered to all participants at baseline and at follow-up after the last consultation within a 14-day time frame. Each participant was registered by the GPs who used a registration form that included symptoms and relevant diagnoses, and the participant's sick leave status at baseline, the last session, and at the end of the 11-week study period.

### Outcomes

The outcome measures were assessed at the individual participant level. The questionnaires used as primary and secondary outcomes are described in detail in the study protocol (Supplementary Material S3). The 11-week timeframe was chosen as it struck a balance between practicality and allowing sufficient time to assess the intervention's impact on sick leave.

### Primary outcome

The Patient Global Impression of Change (PGIC) was utilized as the primary outcome measure of the study, which evaluates changes in clinical status based on patient-reported experiences of changes in function, symptoms, and quality of life from baseline to follow-up, and involved posing one question: "Describe the changes in function, symptoms, and overall quality of life since you received treatment by your GP." Participants were then presented with seven response alternatives, ranging from "very much better" to "very much worse". The brevity and patient-driven nature of the PGIC, along with its capacity to capture both subjective benefits and potential adverse events, and its established validity in chronic pain trials, as demonstrated by prior research e.g.,^19^ contribute to its suitability as the primary outcome measure in our study.

### Secondary outcomes

• The GPs recorded sick leave from the participants’ medical records at baseline, after the final session of the study, and at the 11-week follow-up. At each time point, a participant could either be assigned a value for full time sick leave (yes or no) or a value for partial sick leave. The latter was quantified in percentage points. Both variables were unrelated to the number of hours worked per week. We then defined a joint variable, “sickness absence adjusted for partial sick leave” (SAAPSL), where the scale for full time sick leave was aligned with the percentage scale for partial sick leave (i.e., yes = 100%, no = 0%). Thus, each participant was assigned a value ranging from 0% to 100% for the joint variable. Therefore, "Full-time sick leave" and "Partial sick leave" mentioned in Table 2 are variables that are not further analyzed. Consequently, when we subsequently refer to "sick leave," it specifically pertains to the SAAPSL variable.

**Table 2:** Baseline characteristics by treatment allocation.

Variable	Control (n = 295)^c^	Intervention (n = 238)^c^	Total (n = 533)^c^
Age, mean (sd)	46.9 (12.9)	45.3 (13.2)	46.0 (13.1)
Female	254 (86.1%)	199 (84.0%)	453 (85.2%)
Educational level			
Elementary school (up to 10 years)	32 (11.0%)	27 (11.4%)	59 (11.2%)
High school (10–13 years)	151 (51.9%)	99 (42.0%)	250 (47.4%)
Higher education institutions (>13 years)	108 (37.1%)	110 (46.6%)	218 (41.4%)
Number of children, mean (sd)	2.0 (1.3)	1.7 (1.1)	1.9 (1.2)
Living situation			
Alone	80 (27.1%)	75 (31.9%)	155 (29.3%)
Not alone	215 (72.9%)	160 (68.1%)	375 (70.8%)
**Employment status**			
Long term benefits (>12 months)^a^	153 (55.8%)	168 (71.2%)	321 (62.9%)
Employed	162 (58.3%)	162 (68.4%)	324 (62.9%)
Full time sick leave^b^	55 (24.8%)	54 (33.3%)	109 (34.1%)
Partial sick leave^b^	48 (23.6%)	60 (37.0%)	108 (33.3%)
Partial sick leave in percent^b^, mean (sd)	49.2 (12.7)	50.4 (19.0)	49.9 (16.4)
**Most prominent symptoms**			
Digestive	81 (29.2%)	52 (21.9%)	133 (25.8%)
Musculoskeletal pain	228 (82.3%)	161 (67.7%)	389 (75.5%)
Headaches	154 (55.6%)	125 (52.5%)	279 (54.2%)
Fatigue	205 (74.0%)	203 (85.3%)	408 (79.2%)
Persistent pain (e.g., visceral pain)	172 (62.1%)	90 (38.0%)	262 (51.0%)
**RAND-36**			
Physical functioning, mean (sd)	62.4 (22.9)	67.7 (23.2)	64.7 (23.2)
Role limitations due to physical health, mean (sd)	14.6 (28.9)	20.4 (33.2)	17.2 (31.0)
Role limitations due to emotional problems, mean (sd)	47.7 (44.4)	37.9 (42.7)	43.4 (43.9)
Energy/fatigue, mean (sd)	26.1 (19.1)	24.8 (17.4)	25.5 (18.4)
Emotional well-being, mean (sd)	62.6 (19.5)	56.7 (19.7)	59.9 (19.8)
Social functioning, mean (sd)	47.5 (25.2)	46.3 (26.6)	47.0 (25.8)
Pain, mean (sd)	35.3 (23.4)	41.3 (22.9)	38.0 (23.3)
General health, mean (sd)	37.9 (18.8)	40.9 (19.8)	39.3 (19.3)
**Sick leave**			
Sickness absence adjusted for partial sick leave in percent^b^ (SAAPSL), mean (sd)	49.7 (42.4)	52.0 (41.4)	50.9 (41.8)

The table entries are N (%) for categorical variables and mean (standard deviation) for numerical variables.

a Participants with full or partial long-term benefits due to a disability. A participant can receive long term benefits while being employed. Among the 324 employed participants, some received partial long-term benefits (>50% work disability), with a total of 275 participants (86.2%) reporting that they did. Of those, 129 participants (82%) were in the usual care group and 146 participants (91%) were in the intervention group.

b Only 324 participants who were employed were included in the analysis of sick leave.

c Due to missing values the respective available number of observations for most variables were in the range 274–295, 235–238 and 510–532 for the control, intervention and total. The two variables Age and Number of children had 441 and 369 observations, respectively.
• Work-related self-efficacy, which was assessed by the Return-to-Work Self-efficacy Questionnaire (RTW-SE). This is a validated 11-item questionnaire that captures a person's self-efficacy with a specific focus on the return to work process.^20^
• Health-related quality of life (HRQoL) was assessed using the RAND-36, a 36-item measure that evaluates eight aspects of health, including physical functioning, role limitations caused by physical and emotional problems, social functioning, emotional well-being, energy/fatigue, pain, and general health perceptions.^21^ A difference of 3–5 points between groups in the RAND-36 score is considered clinically relevant.^22^
• Patient experiences with the intervention. For that purpose we utilized The Patient Experience Questionnaire (PEQ) who was developed in primary healthcare settings to evaluate participants' experiences with the consultation.^23^ In our study, we utilized a shortened version of the PEQ.
• Baseline characteristics of the participants: age, gender, educational level, number of children, living alone/not living alone, receiving long term benefits, being employed, and being on sick leave (full time or part time).
• Baseline characteristics of the GPs: age, gender, specialist/not specialist, and the number of patients they are responsible for at their GP office.

### Statistical analysis

An initial power calculation was based on a 7-point scale, where 1 represented "very much better", 4 was unchanged and 7 was "very much worse". Although we found no previous studies on using PGIC to assess patients with MUPS specifically, a study on chronic pain patients and their PGIC scores after 12 months was utilized.^19^ We used a group-randomized trial calculator [https://researchmethodsresources.nih.gov/methods/grt], where the intervention group's score would shift from 4.2 to 3.5 from before to after the intervention, while the control group’s score would remain constant. Additionally, we assumed that the score would have a standard deviation of 1.36. The type I error rate was set to 5%, and the power to 90%. To account for clustering on the GP level, we set the mean number of patients per GP as low as 3 and assumed an intraclass correlation coefficient (ICC) equal to 0.10. The power calculation results suggested that a total of 66 GPs (33 in both the intervention and control groups), which is approximately 200 patients, would be sufficient. To account for drop-out and missing responses, we aimed to recruit 100 GPs.

The primary outcome was PGIC analyzed using a Wilcoxon Rank-Sum test and t-tests for means. To estimate the effects of the intervention, we used mixed models’ linear regression. The PGIC and PEQ outcomes were only observed at the end of follow-up, so we estimated the intervention effect using the coefficient of a dichotomous variable for group (control = 0, intervention = 1), with random intercepts on the GP level included. For the other outcome variables sick leave, RAND-36, and RTW-SE, which were observed at both baseline and follow-up, we estimated the intervention effect using the coefficient of the interaction between time (baseline = 0, follow-up = 1) and group. All patients observed once or twice were included, and random intercepts on the patient level were added. The unadjusted models included only the variables and random intercepts mentioned above, while the adjusted models also included baseline patient characteristics. We performed all statistical analyses using Stata version 17.

### Ethical approval

We followed the guidelines outlined in the Declaration of Helsinki^24^ to ensure ethical conduct during our study. Prior to enrollment, all prospective participants were notified of their right to decline participation without incurring any negative consequences. Ethical restrictions prevented registration of the number and reason of participants who chose not to participate. Given that patients with MUPS may be considered a vulnerable population, we acknowledge that it may have been difficult for them to decline their physician's invitation to participate. Nevertheless, we believe that the potential benefits of investigating more effective tools to better aid patients with MUPS in primary care outweigh the potential risks.

The Regional Ethics committee (reference number 387480) and the Norwegian Centre for Research Data (reference number 675741) approved our study.

### Declaration of generative AI and AI-assisted technologies in the writing process

During the preparation of this work the authors used ChatGPT in order to enhance language and readability. After using this tool, the authors reviewed and edited the content as needed and take full responsibility for the content of the publication.

### Role of the funding source

The funder of the study had no role in study design, data collection, data analysis, data interpretation, or writing of the report.

## Results

**Fig. 1:**
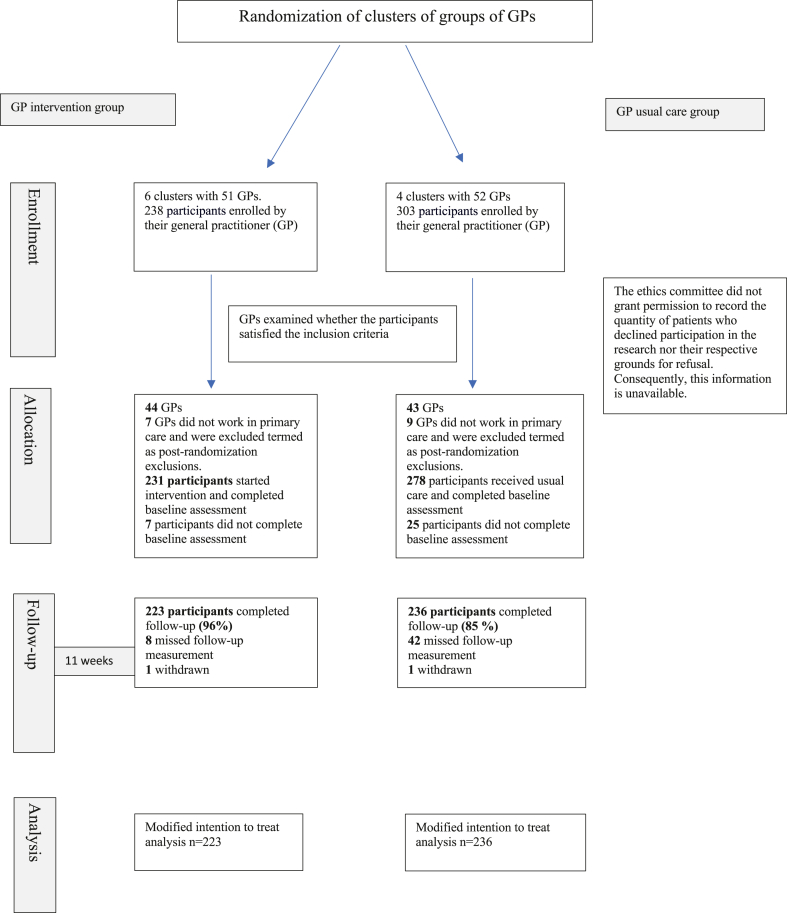
**The flow of participants through thestudy.**

Recruitment took place between March 7 and April 1, 2022. Both intervention and usual care GPs recruited 1–10 patients each. Fig. 1 illustrates the flow of participants through the study. A total of 541 participants with MUPS were included by 103 recruited GPs, of which 238 received treatment with the ICIT and 303 received usual care. However, 16 GPs were excluded from the study due to their transition out of primary care at the time of recruitment, which we refer to as post-randomization exclusions. The GP characteristics are described in the Supplementary Material (Supplementary Table S2).

The baseline questionnaires were completed by 231 participants in the intervention group and 278 participants in the usual care group. The follow-up form was completed for 223 participants (96%) of the intervention group and 236 participants (85%) in the usual care group.

Table 2 provides socio-demographic and clinical baseline characteristics of participants: Age, gender, educational level, number of children, living situation, employment status, sick leave status, and long-term benefits. The mean age of the sample was 46 years (SD = 13.1), with most female patients (85%). More usual care participants had completed high school (52%) compared to the intervention group (42%), while a higher proportion of intervention group participants had completed higher education (>13 years) (47%) compared to usual care (37%).

Participants reported various MUPS symptoms, including digestive, musculoskeletal pain, headaches, fatigue, and persistent pain (e.g., visceral pain), as well as psychological (n = 34), neurological (n = 4), globulus symptoms/tinnitus (n = 7), and non-cardiac chest pain (n = 5). The most common symptoms reported were fatigue (79%) and musculoskeletal pain (76%). Musculoskeletal pain was more prevalent in the usual care group (82%) compared to the intervention group (68%), while fatigue was more prominent in the intervention group (85%) compared to usual care (74%).

### Primary outcome

**Fig. 2:**
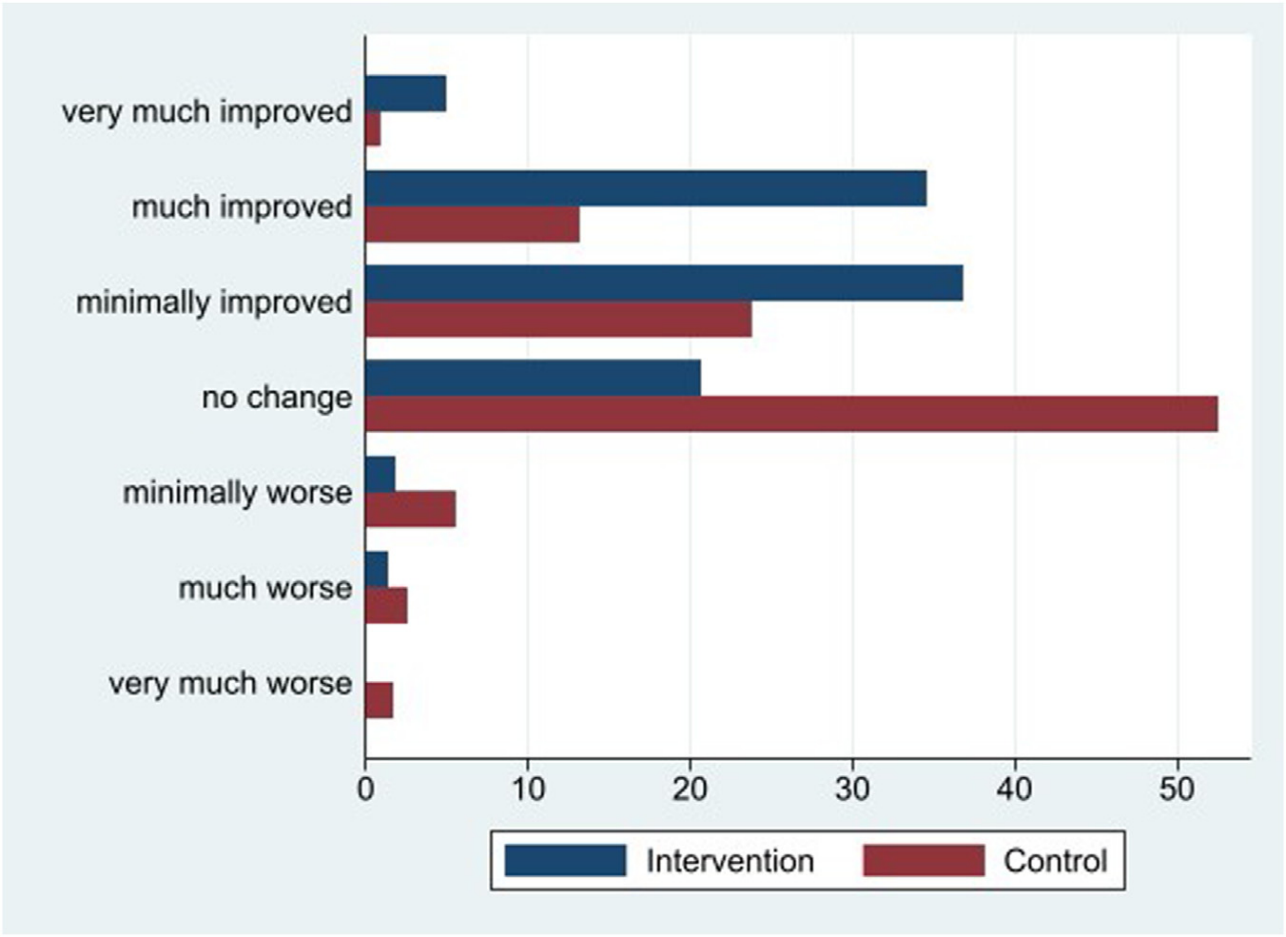
**Patient Global Impression of Change at follow-up, inpercent.**

The studies primary outcome was any changes in the participants' functioning, symptoms, and quality of life assessed by the PGIC scale. The PGIC results indicate a substantial difference between the groups, with an estimated difference of −0.8. This difference is considered clinically relevant, as highlighted in Fig. 2, where the percentage breakdown is illustrated. For example, only 21% of participants in the intervention group reported "no change" as compared to 53% in the usual care group. Further, 76% in the intervention group reported an improvement compared to 38% in the usual care group. This stark contrast further supports the clinical relevance of the intervention.

We calculated the mean values for the PGIC scores and observed a statistically significant difference between the intervention and usual care groups (p < 0.0001). The mean PGIC value for the usual care group participants (n = 236) was 3.6 [95% CI 3.5–3.8] while the intervention group (n = 223) had a mean value of 2.8 [95% CI 2.7–3.0] with respect to the PGIC scores.

The mixed model regressions presented in Table 3 yielded similar reductions; −0.8 [95% CI −1 to −0.6] in the unadjusted model and −0.8 [95% CI −1.0 to −0.5.] in the adjusted model.

**Table 3:** Estimated effects of the intervention on primary and secondary outcomes, coefficients from mixed model linear regressions.

Dependent variables	Unadjusted			Adjusted		
	Coef.	95% CI	p-value	Coef.	95% CI	p-value
**Primary outcome**						
Patient Global Impression of Change^a^	−0.8	(−1.0 to −0.6)	<0.001	−0.8	(−1.0 to −0.5)	<0.001
**Secondary outcomes**						
Sick leave^b^						
Sickness absence adjusted for partial sick leave (SAAPSL) in percent	−23.6	(−30.2 to −17.0)	<0.001	−24.9	(−34.1 to −15.7)	<0.001
Health-related quality of life (Rand-36)^b^						
Physical functioning	2.9	(0.4–5.3)	0.022	3.6	(0.4–6.9)	0.029
Role limitations due to physical health	9.7	(3.7–15.7)	0.001	8.3	(0.3–16.3)	0.043
Role limitations due to emotional problems	6.2	(−1.5 to 13.9)	0.113	9.8	(−0.5 to 20.1)	0.061
Energy/fatigue	7.1	(3.9–10.3)	<0.001	6.2	(1.9–10.4)	0.005
Emotional well-being	3.4	(0.7–6.0)	0.014	3.5	(0.1–7.0)	0.045
Social functioning	7.1	(3.4–10.8)	<0.001	8.6	(3.7–13.4)	0.001
Pain	5.7	(2.6–8.9)	<0.001	5.9	(1.8–10.1)	0.005
General health	5.0	(2.7–7.2)	<0.001	4.3	(1.4–7.3)	0.004
Patient Experience Questionnaire (PEQ)^a^						
Communication	0.2	(0.0–0.3)	0.026	0.2	(0.0–0.4)	0.019
Barriers	0.0	(−0.1 to 0.1)	0.920	−0.1	(−0.2 to 0.1)	0.411
Emotions	0.4	(0.2–0.7)	<0.001	0.5	(0.2–0.8)	0.002
Work related self-efficacy^b^	0.2	(0.0–0.4)	0.113	0.4	(0.0–0.7)	0.026

Regression coefficients (Coef.) and corresponding 95% confidence intervals (95% CI).

The adjusted regressions included the following independent variables at the patient level: age, female, education level, number of children, alone, long-term benefits, employed, and full-time sick leave. The latter three variables, which are work-related, were omitted in the adjusted regression for partial sick leave. Confer the supplement for intraclass correlation (Supplementary Table S1).

a Patients observed at end of follow-up only.

b Patients observed at baseline and end of follow-up.

### Secondary outcomes

#### Sick leave

**Fig. 3:**
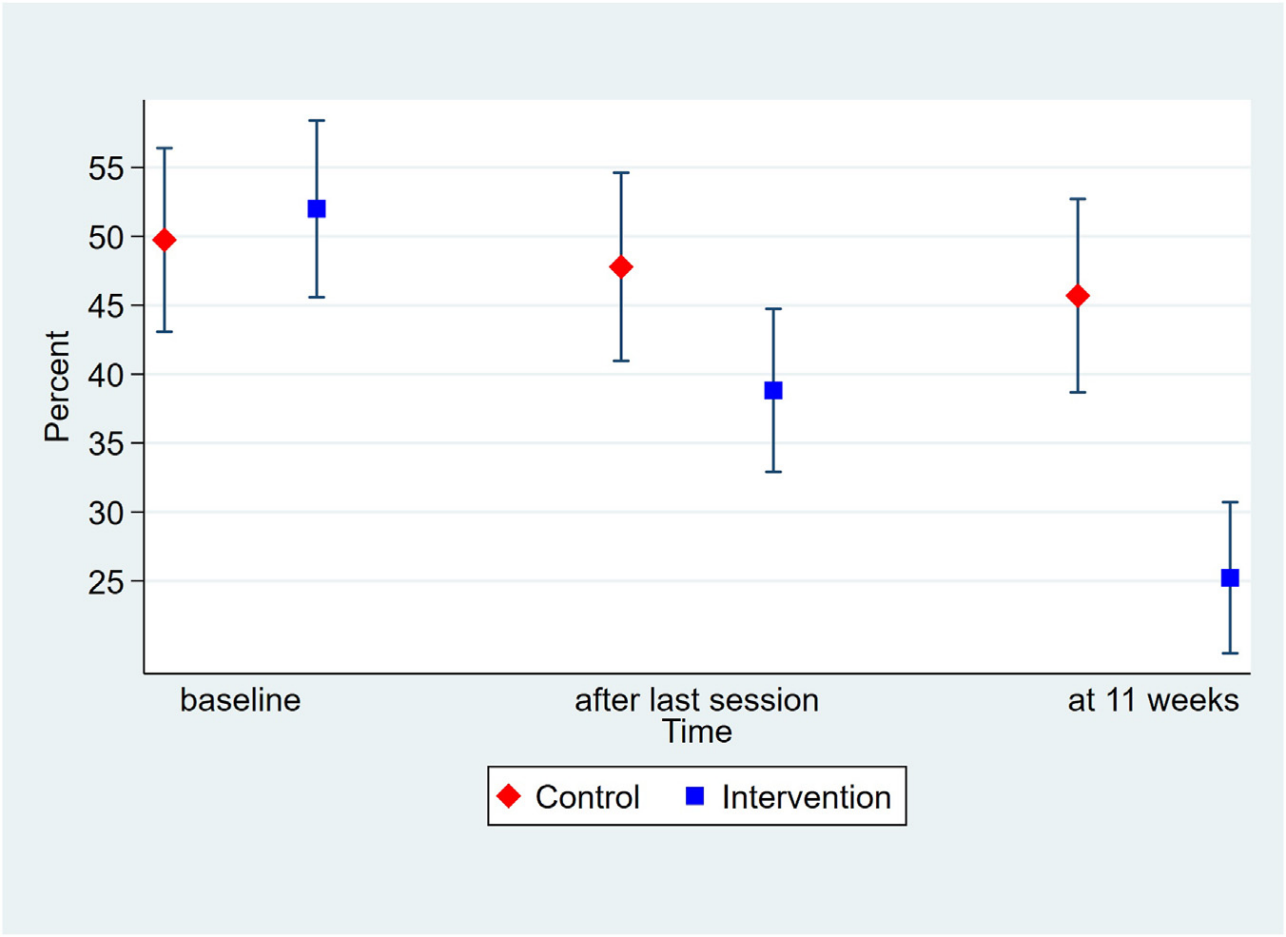
**Sickness absence adjusted for partial sick leave (SAAPSL) at baseline and follow-up.** Means with 95% confidence intervals, estimated separately and unadjusted.

The current study aimed to investigate sick leave among participants employed in both the usual care group (n = 162) and the intervention group (n = 162). The analysis of sick leave (SAAPSL) was graphically presented in Fig. 3, which demonstrates a decrease in sick leave for participants in the intervention group compared to the usual care group from baseline to follow-up. At the 11-week mark, we observed a significant difference in sick leave between the intervention group and the usual care group (two sample t-test, p < 0.001. The intervention exhibited a substantial 27-percentage point decrease in sick leave, reducing it from 52.0% to 25.2%. In contrast, the usual care group experienced a smaller 4-percentage point decrease, bringing their sick leave from 49.7% to 45.7%. Table 3 displays the estimated effect of the intervention, which was −24 percentage points ([95% CI −30.2 to −17.0]; p < 0.001) and −25 percentage points ([95% CI −34.0 to −15.8]; p < 0.001) in the unadjusted and adjusted mixed model regressions, respectively.

#### Work-related self-efficacy

The study evaluated work-related self-efficacy at baseline and follow-up for all participants in the study. The results indicate that the intervention group had higher estimates of work-related self-efficacy compared to the usual care group. These differences were statistically significant in the adjusted model. Specifically, while the unadjusted model showed a mean difference of 0.2 ([95% CI −0.0 to 0.4], p = 0.113), the adjusted model demonstrated a mean difference of 0.4 ([95% CI −0.0 to 0.7], p = 0.026), as presented in Table 3.

#### Health-related quality of life

Table 3 illustrates the results of Rand-36 in assessing health-related quality of life. Specifically, for the Physical Functioning subscale of Rand-36, the mean difference was 2.9 ([95% CI 0.4–5.3], p = 0.022) in the unadjusted model and 3.6 ([95% CI 0.4–6.9, p = 0.029) in the adjusted model. The results thus demonstrated a significant clinical and statistical enhancement in symptoms across seven of the eight items in the intervention group compared to usual care, which included physical functioning, role limitations due to physical health, energy/fatigue, emotional well-being, social functioning, pain, and general health.

#### Patient experiences with the intervention

To assess participants' experiences during their consultations, we utilized a shortened version of the Patient Experience Questionnaire (PEQ).^23^ Our analysis revealed that, in comparison to the usual care group, those in the intervention group reported a better overall experience during their consultations. Specifically, they reported more effective use of time, productive conversations, and high levels of confidence in their GPs. The intervention group reported feeling well-cared for, receiving adequate help, and understanding from their GPs. They experienced less worry and more positive affect than the usual care group, feeling strengthened, cheerful, and relaxed. In addition, the participants in the intervention group reported effective time utilization, productive conversations, and receiving adequate help. Offering reassurance to affected patients is challenging due to the lack of a clear medical explanation.^25^ Therefore, we deem our findings regarding the participants' experience with the intervention to be not only statistically significant but also clinically relevant.

We calculated the mean difference between the intervention and usual care groups and found that for the PEQ communication and emotional factors, the difference between the groups was statistically significant. Table 3 presents the results of PEQ, and for the communication factor, the mean difference was 0.2 ([95% CI 0.0–0.3], p = 0.026) in the unadjusted model, and 0.2 ([95% CI 0.0–0.4], p = 0.019) in the adjusted model.

Although GPs were instructed to monitor and report any potential side effects or serious adverse events, we did not include a comprehensive assessment of side effects in our study.

## Discussion

The current pragmatic cluster randomized controlled trial demonstrates significant effects of a structured communication tool (ICIT) used by GPs in primary care for patients with MUPS across multiple domains. Compared to usual care, the participants in the intervention group reported statistically and clinically significant improvements in function, symptoms, quality of life, reduced sick leave, increased work-related self-efficacy, and greater satisfaction with the communication during the consultations. Our findings further refute the idea that participants felt coerced by their GP to return to work, as they expressed significantly higher satisfaction with communication during the consultations compared to the usual care group. High compliance rates were observed in both groups (96% for intervention and 85% for usual care), which enhances external validity. Moreover, ICIT is a novel work-focused communication tool for use in primary care that builds on CBT which is an effective treatment for anxiety and depression.^26^

Following the conclusion of the study, there has been a notable surge in enrollment among GPs for courses aimed at enhancing their proficiency in utilizing the structured communication tool, ICIT.

The enrollment figures have surpassed 340 GPs within a one-year timeframe, underscoring the interest demonstrated by GPs in acquiring skills pertaining to this communication tool. Moreover, this high level of engagement suggests that the communication tool's applicability extends beyond a select group of highly interested GPs.

Although this study has some strengths, it is crucial to acknowledge certain limitations. Firstly, not being able to blind both participants and GPs to intervention allocation poses challenges in excluding the possibility of placebo or Hawthorne effects. Secondly, using participants' medical records as the primary method of registration by GPs may introduce potential bias in the sick leave attendance data. Thirdly, the absence of a clear consensus on the definition of MUPS across studies may limit transferability of results across different cultural and national contexts. Although a cluster randomization was used to maintain intervention fidelity and minimize contamination, randomizing individual participants would have been preferable to improve the study's internal validity. Fourthly, we acknowledge that the potential promotion of presenteeism inherent in the intervention should be further investigated. While our study did not include detailed measures specifically targeting presenteeism, we recognize the importance of assessing this aspect in future studies. Fifthly, the follow-up period of this study was 11 weeks, which could have been longer to increase validity and detect long-term effects. However, the sick leave taken by patients with MUPS acts as a progressive barrier to return to work^27^; 14% of patients who are absent from work for more than 13 weeks, never return to work.^28^ We therefore argue that the 11-week follow-up period is sufficient to demonstrate the clinical relevance, given the potential long-term consequences of prolonged sick leave on patients' ability to return to work successfully.

The study adhered to the established protocol. Unfortunately, a regrettable oversight occurred as the trial registration was not updated before commencement, leading to a minor discrepancy between the trial registration and the manuscript. Our study is further limited by the unavailability of recorded data on participants who declined to participate and the reasons for their non-participation, owing to ethical constraints. However, since the enrollment process was efficiently executed, we believe that the GPs identification of participants who met the study's inclusion and exclusion criteria was straight forward. Furthermore, the study's external validity is further reinforced by the inclusion of a significant number of participants. Nevertheless, it is essential to recognize that a large sample size does not render the study immune to selection bias. The study participants presented with significant complaints and willingly sought help, which enhances the relevance of the findings to the target population. To preempt potential participant disappointment and influence on study outcomes, GPs in the usual care group received ICIT training after the study's conclusion on May 21, 2022. This proactive step was taken to address potential participant letdown and likely served to strengthen the observed effects of ICIT.

Finally, it is worth noting that our study was carried out in Norway, where all citizens have access to their regular GP. Thus, the generalizability of our findings to countries with dissimilar healthcare structures may be limited.

Research indicates that CBT interventions are effective for MUPS patients in specialized settings, but less effective in primary care.^3^^,^^6^ GP-led therapy has been recommended for interventions like ICIT.^29^ An effect evaluation of an intervention to reduce sick leave found that patients with common mental disorders returned to work faster after undergoing work-focused CBT, which supports the use of a work-oriented approach applied in the communication tool ICIT.^11^

In line with another study examining the impact of a brief CBT intervention administered by nurses to patients with MUPS,^30^ our study similarly demonstrated an improvement in physical functioning and a reduction in pain. Unlike the nurse-led study, our study showed significant improvements in emotional well-being, energy, social functioning, and overall health. We found statistically and clinically significant improvements in seven out of eight measures, as evidenced by a >3 point difference between groups in the RAND-36 score.^22^ Our study's notable strength is the GP setting, and the transdiagnostic communication tool ICIT, which suggests broader implications for other medical conditions as well.^31^

## Contributor

The study was conceptualized by ELW, SER, ML, and CA, who also secured funding for the project. ELW, SER, ML, and CA drafted the study protocol, while CA was responsible for designing and delivering the intervention. Data were collected by the participating GPs. KRW, ELW, SER, ML, and CA collaborated on writing the statistical analysis plan, and KRW performed the statistical analyses. CA drafted the manuscript, which was subsequently reviewed and critically revised by all authors. All authors have read and approved the final manuscript. The corresponding author confirms that all authors meet the criteria for authorship and that no eligible individuals have been excluded.

## Data sharing statement

An anonymized dataset will be made available upon reasonable request, and after approvals have been obtained from relevant bodies.

## Declaration of interests

All authors have completed the ICMJE uniform disclosure form at www.icmje.org/disclosure-of-interest/.

The first author, who serves as the guarantor for this manuscript, confirms that the study being reported is presented with honesty, accuracy, and transparency. No critical aspects of the study have been omitted, and any deviations from the planned study design, including those registered, have been duly explained.

The first author of the study (CA) has developed the innovative communication tool ICIT. Following the culmination of the research on May 2022, two courses were arranged to train GPs on how to utilize ICIT. Notably, CA, together with SER, ML and ELW, were among the lecturers who imparted knowledge during these courses and were economic compensated for their efforts with standard fee. It is essential to emphasize that there are no financial benefits or royalties associated with the use of ICIT as a communication tool.
