# Supplementary material for: The effects of a structured communication tool in patients with medically unexplained physical symptoms: a cluster randomized trial
Source: eClinicalMedicine. 2023 Oct 6;65:102262. doi: 10.1016/j.eclinm.2023.102262 (PMC10579279; doi:10.1016/j.eclinm.2023.102262)
Supplement: Supplementary Material [file mmc1.pdf]

SUPPLEMENTARY MATERIAL

Cathrine Abrahamsen

Specialist in family medicine. PhD student at Department of General Practice, Institute of Health and Society at the University of Oslo.

Supplementary material

**Supplementary material 1**:<br>• The Background and development of the structured communication tool Individual Challenge Inventory Tool (ICIT)	p 1-15
**Supplementary material 2**:<br>• The structured communication tool ICIT complete version	p 16-19
**Supplementary material 3:**<br>• Study protocol	p 20-25
**Supplementary material 4:**<br>• Table S1. Intraclass correlation, supplement to mixed model linear regressions reported in Table 3	p 26
**Supplementary material 5:**<br>• The structured communication tool ICIT - search strategies	p 27-28
**Supplementary material 6:**<br>• Table S2. General practitioner characteristics by treatment allocation	p 29

Supplementary material 1

The Background and development of the structured communication tool Individual Challenge Inventory Tool (ICIT)

I am a 49-year-old general practitioner (GP) with 20 years of experience in primary care. Over the course of my career, I have tried to help my patients to navigate their health challenges. Throughout my career, I have found immense fulfillment in assisting patients in managing their health challenges. As a GP, I have developed an interest in the role of effective communication in enhancing patients' coping strategies and overall well-being.

Patients with Medically Unexplained Physical Symptoms

Patients with Medically Unexplained Physical Symptoms (MUPS) encompass persistent bodily symptoms and functional impairment that lack an identifiable medical condition or pathology as an explanation. This condition can be a source of frustration for both patients and physicians alike. Individuals experiencing MUPS often express dissatisfaction with their medical treatment, perceive stigmatization, and may feel that their concerns are not taken seriously [1].In the management of patients with MUPS, the role of the GP is pivotal due to the necessity of providing long-term follow-up care. It is estimated that up to 40% of all primary care consultations involve patients presenting with MUPS [2,3]. Moreover, the prevalence of MUPS is notable in specialist healthcare settings as well, owing to the high number of referrals and subsequent investigations[2].

Patients with MUPS frequently encounter psychological distress, social isolation, and a diminished quality of life, ultimately resulting in increased healthcare utilization and expenses associated with sick leave [3-5].Nevertheless, patients with MUPS often face marginalization and may turn to their healthcare providers to request sick leave when they feel incapable of managing to stay at work. With a dedicated focus on fostering resilience, I have made it a priority to assist these patients in maintaining their occupational engagement whenever possible. However, I have noticed that prescribing sick leave frequently falls short of achieving the desired improvement for these individuals. In some cases, discussions arise where patients attempt to validate their illness, potentially unintentionally exaggerating their functional limitations. This has led me to contemplate an alternative approach - one that centers around identifying what remains feasible for the patient within their work environment, despite their symptoms.

What was the purpose of a structured conversation tool?

Recognizing the need for a communication tool that shifts the focus from problems to possibilities, I sought to develop a structured conversation tool that enhances patients' coping abilities and resilience in patients with MUPS.

This tool should also serve the purpose of assessing the suitability of sick leave as a treatment option for each patient, considering their individual circumstances. Through the utilization of this conversational tool, my objective is to cultivate a patient-centered approach that empowers individuals to actively engage in their daily lives, despite their ailments. By shifting the focus towards what is still achievable, rather than dwelling on limitations, this tool was meant to promotes patients' involvement in decision-making regarding their work participation. By emphasizing possibilities, it aims to inspire patients to explore alternative strategies, identify feasible adjustments, and develop the necessary skills to effectively manage their symptoms while remaining actively involved in the workforce.

How could this be achieved in a regular primary care consultation?

During a one-year course in cognitive therapy, I became acquainted with the "problem list" tool, which I have subsequently refined and adapted for implementation in primary care consultations, irrespective of specific diagnoses. Recognizing the significant role that GPs play in the management of patients with MUPS, I have tailored this tool to suit their specific needs.
The conversation tool developed for this purpose is named "Individual Challenge Inventory Tool" (ICIT) - aimed at promoting patient self-care and assisting physicians in assessing the need for sick leave. The structured communication tool ICIT intended to empower patients and facilitate a shared understanding between the patient and the GP concerning the origins and implications of non-specific health concerns. The ICIT guides the conversation as a treatment manual from "welcome" to "gooby" enabling a systematic exploration of the patient's challenges and goals. By utilizing the ICIT, the GP can effectively engage the patient in a collaborative dialogue, promoting patient autonomy regarding their self-care, and assessing the need for sick leave if necessary.

Patients with MUPS often exhibit resistance towards psychological interventions as they may not perceive their symptoms as originating from psychological factors [6]. However, by offering integrated treatment, as also suggested in the literature [7], through the utilization of the structured communication tool ICIT, GPs have the potential to enhance the likelihood of treatment acceptance among this patient group.

What was the purpose and documentation of The MUPS explanatory model?

The literature suggests that patients with MUPS needs help to accept their symptoms, provide coping strategies, and create a positive treatment expectation [8]. However, there is limited research on how this should be achieved. Additionally, it can be difficult for doctors to provide a reassuring explanation to patients with MUPS due to the lack of a clear medical cause [9]. To provide effective assistance, it is crucial for both the GP and the patient to establish a shared understanding of the patients' ailments. In order to achieve this mutual understanding of the patient's symptoms, I have developed a "MUPS explanatory model" (figure 1) that draws upon "The cognitive activation theory of stress" [10] ,the concept of allostatic overload [11], and how allostatic overload is detectable through autonomic, hormonal, immunological, and genetic measures [12]. Prolonged excessive strain on the human body's physiological adaptability can have detrimental effects on health. This phenomenon, known as "allostatic overload" [12-14], occurs when persistent and/or intense threats to an individual's well-being and integrity eventually deplete the body's capacity for flexibility. Notably, stresses that are commonly considered physical in our culture (such as under/over/malnutrition, pollution, lack of sleep, infections, and noise) have been found to impact the body at the

same physiological "level" as stresses traditionally regarded as psychological (such as a life marked by threats, neglect, abuse, or overwhelming caregiving responsibilities). Both forms of stress can contribute to various consequences, including autonomic dysfunction, alterations in the immune system, chronic low-grade inflammation, disturbances in hormonal regulation, and accelerated cellular aging, as evidenced by telomere length measurements[12]. The MUPS explanatory model serves as a framework for explaining how prolonged stress, irrespective of whether it is perceived as physical or psychological, continues to activate the body's stress response, leading to a wide range of symptoms and health conditions.

How the MUPS explanatory model is used in the structured communication tool ICIT

The GPs are guided to treat patients with MUPS in the following way (p 16-17 in the extended version of the structured communication too ICIT):

Step-by-step approach in patients with medically unexplained physical symptoms:

"To help you with your health concerns, we'll be using a step-by-step approach. Firstly, we'll summarize what we have discussed thus far, then we'll collaborate to find a possible explanation for your symptoms, and lastly, we'll develop a plan to help improve your wellbeing. To achieve this effectively, we'll require scheduling three appointments soon. Does this plan work for you?"

3a: CONSULTATION NUMBER 1

"During our first consultation, let's review the tests and findings we've gathered so far."

If possible, try to explain for the patient their condition for example non-cardiac chest pain, fibromyalgia, tension headaches or tinnitus.

The GP may say:

- *"It's important to recognize that the body and mind are connected, especially in cases like this. For instance, our heart rate increases when we feel afraid, and it slows down once the perceived danger has passed. This is normal.*
- *"I want you to know that I believe you, and it's not uncommon for doctors to be unable to determine the underlying cause of health issues through blood tests or other medical exams. For instance, after an infection, it's typical to feel exhausted and sluggish, even after the infection has resolved."*
- *"Fortunately, ongoing research is exploring how we can provide effective treatments for patients in your condition. There is help available, and it is possible for you to improve."*
- *"Lastly, do you have any ideas or thoughts on what might be causing your symptoms?*
- *"Are there any patterns or connections you've noticed? Let's discuss this further during our next session."*

3b: CONSULTATION NUMBER 2

- Use the MUPS explanatory model (figure 1) to create a shared understanding of the symptoms:

"We've learned a lot about what might be causing your symptoms, and I have a visual model that can help us understand this together. Would you be okay with me showing it to you?"

- Provide the patient with their own copy of the model in a printed handout.
- "What triggers long-term stress in your life?" Identifying maintaining factors is a part of the treatment process. Ask the patient to complete either the "problem list" or the "list of opportunity " on the MUPS explanatory model, which we'll discuss in our next session.

- It's important not to jump into implementing solutions during consultation no. 2. The patient needs time to digest the MUPS explanatory model, and it's essential for them to reflect on what they believe is sustaining long-term stress in their life. By refraining from implementing solutions at this stage, we can give the patient the space and time they need to fully engage with the process of understanding their symptoms and identifying potential solutions.

3c: CONSULTATION NUMBER 3: ICIT

Consultation no. 3 is the ICIT action phase of the consultation. It's important to start this phase promptly to utilize the time effectively. The following is a list of interventions that the doctor can use based on the patient's situation. The aim is to develop a short-written plan using either «*list of opportunity,", "problem list"* or *"job list",* which may be combined depending on the patient's needs.

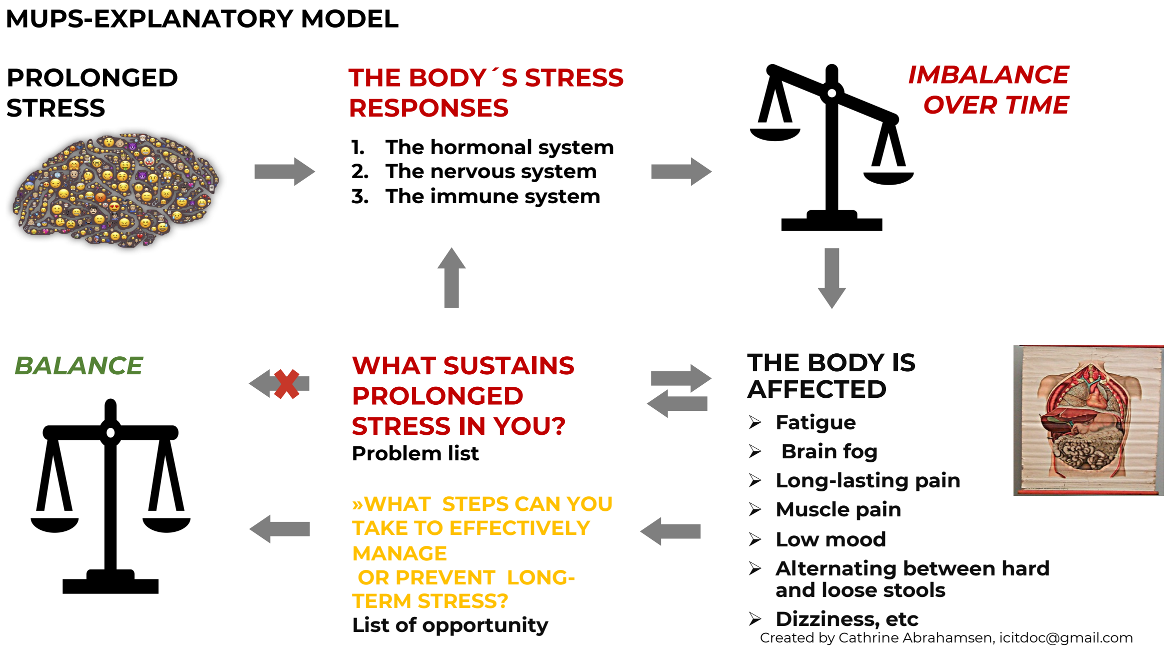

Figure 1: MUPS explanatory model

Why a structured communication tool using Socratic dialogue?

During consultations with patients with MUPS, I frequently encountered challenges in knowing how to effectively communicate with them. I consistently sought out impactful phrases and expressions that could provide support and assistance to these patients. To address this challenge, I began incorporating open-ended questions knows as Socratic questions into my conversations with patients, focusing on exploring what tasks or responsibilities they felt capable of performing despite their complaints. If patients found these Socratic questions to be beneficial, I made it a point to record them in a dedicated notebook. I found that reusing these questions in subsequent conversations

with other patients proved valuable. As a result, my notebook became filled with helpful Socratic questions that patients found supportive and meaningful. These Socratic questions were subsequently organized into themes, forming the foundation of an activity plan. The themes included: 1) "Problem list" for patients who felt overwhelmed by various issues, 2) "List of opportunity" for patients who are experiencing fatigue and a lack of effective coping mechanisms in their daily lives, and 3) "Job list" for cases where evaluating the need for sick leave was a prominent topic during the consultation. Figure 2 illustrates the structure within the structured communication tool ICIT, showcasing the organization and framework of these themes. The condensed version of the structured communication tool ICIT, serving as a manual during encounters with GPs are shown in figure 3.

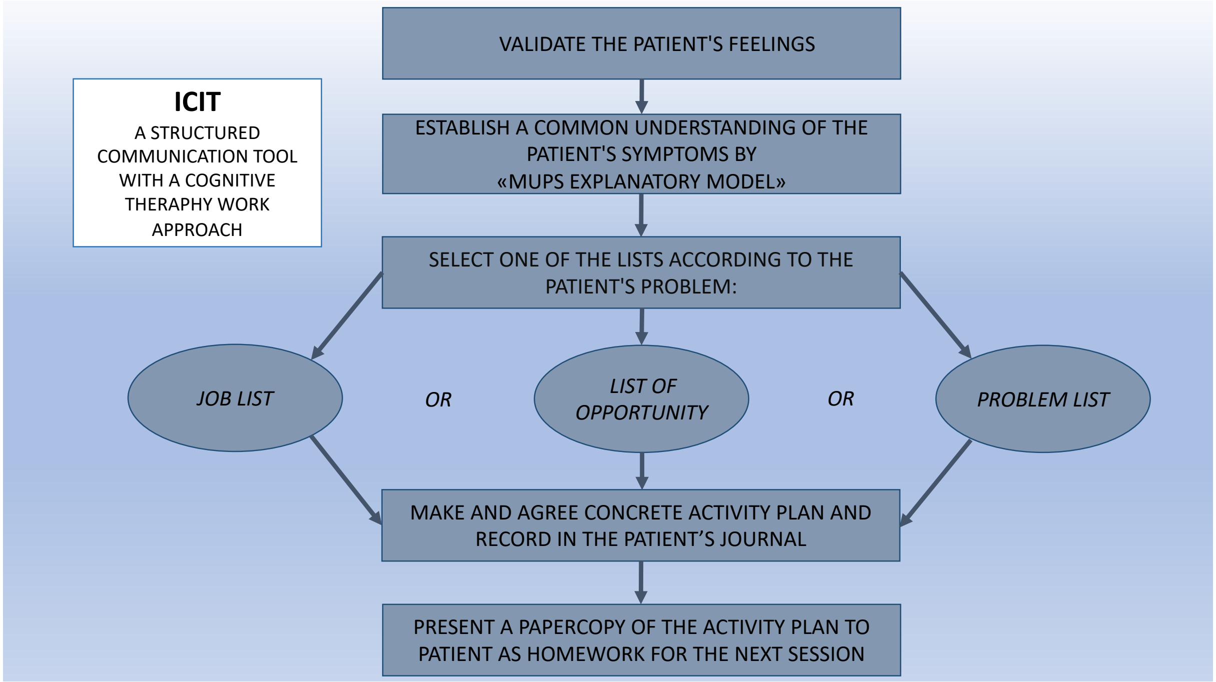

Figure 2: The structure in the structured communication tool ICIT

ICIT-A STRUCTURED COMMUNICATION TOOL WITH A COGNITIVE THERAPY WORK APPROACH

1.SET AGENDA for the session
Initiate the session by greeting the patient courteously, expressing apologies for any delays, engaging in casual conversation, and collaboratively establishing the agenda by asking: "*What topics would you like to cover*?" and "*is there anything else you would like to discuss?*"

2.VALIDATION - Confirm the patient's feelings

- Validate the patient's feelings with praise: *"I'm glad you came today. It's clear that you don't want to feel this way."*
- Acknowledge the patient's situation: *"It's understandable that you feel tired given your condition. It's not how you would like things to be."*
- Legitimize the patient's experience: *"It's completely normal to feel fatigued and exhausted when you've been experiencing pain and discomfort for a prolonged period."*
- Confirm the patient's feelings: *"I understand that your pain/headache/digestive problems can be challenging, and I would like to gain a better understanding of how they impact your daily life."*
- For patients with MUPS: *"Research indicates that persistent stress can have a significant impact on your body, contributing to the symptoms you are experiencing."*
- Provide support and insight: *"I would like to offer you a method that may assist with managing your symptoms. Would you be open to trying it together?"*

Choose one of the following lists depending on the patient's problem.

3a. JOB LIST
Taking sick leave =treatment.
However, it's worth noting that being at work may have positive effects on your health.

- Consider asking the patient exploratory questions to help them identify positive aspects of work and areas that could be improved, such as:

• *"What aspects of work would be helpful for you given your current situation?"*
•*" Can you identify something positive about your work despite your symptoms?"*
• *"Are there specific tasks or aspects of work that bring you joy or satisfaction?"*
•*" Are there any tasks that are particularly challenging right now, and that you would benefit from delegating or seeking support for?"*

- Create a specific and detailed list in the patient's medical record outlining the accommodations that could assist them in managing their symptoms while at work.
- This job list can serve as a basis for discussing potential adjustments or facilitation with their employer.

3b. PROBLEM LIST

"To better assist you, I would like to get an overview of all the issues you feel are important, without delving into the details immediately. It's akin to a game of cards, where we need to distribute all the cards before we can start playing. Once we have a full picture, we can work together to address the issues at hand."

Review the points with the patient and ask:

"What are the problems that you can currently control, and which ones are beyond your control?"

Encourage the patient to select a problem that they believe is solvable. Write down step-by-step actions that can be taken to address this problem, including:

"What has helped in similar situations in the past?"
"Is there someone who can assist you with this?"

By using this approach, we can work collaboratively to address the patient's concerns and develop a plan of action that is both realistic and effective.

3c. LIST OF OPPORTUNITY
"A list of opportunity" can be helpful for patients who are struggling with a lack of motivation, feelings of helplessness, and social isolation. Some useful prompts for this list include:

- *"I'd like to understand how these problems are affecting your life. Is there anything that typically brings you joy that you find difficult to do now?"*
- *"Are there any activities that you usually enjoy that you still find fulfilling?"*
- *"What activities do you find restful and revitalizing? And is it beneficial to have a structured approach to resting, including when and how you rest?"*
- *"In what ways can your partner, friends, or family provide support that would be helpful given your current situation?"*

Using this "list of opportunity «can help patients identify opportunities for positive change and develop a sense of control in their lives."
To help patients prioritize and plan their next steps, create a bullet-pointed list of potential actions, and ask:
'Which of these actions do you feel would be the easiest for you to start with?'
Once a particular action has been identified, create a bullet-pointed plan for how it can be carried out. Then, ask the patient:
'How can we make this plan more feasible for you?'
By involving the patient in the process of identifying and planning actions that feel manageable and realistic, we can help them build a sense of competence and momentum towards positive change."

4.ACTIVITY PLAN
Collaborate with the patient to create a specific and actionable activity plan and document it in their medical record. Provide the patient with a hard copy of the plan to review as homework before the next session.

5.FOLLOW-UP in a new consultation: Review the homework.

Created by *Cathrine Abrahamsen icitdoc@gmail.com*

Figure 3: The condensed version of the structured communication tool ICIT, serving as a manual during encounters with General Practitioners (GPs).

Scientific background of the ICIT

In the following section, I will provide a description of the essence of the structured communication tool ICIT and its scientific background. Cognitive Behavioral Therapy (CBT) is an active, goal-oriented, and time-limited therapeutic approach that targets the reduction of emotional distress and the enhancement of adaptive functioning in patients. CBT encompasses a range of treatment strategies, such as cognitive restructuring, behavioral activation, exposure therapy, and problem-solving techniques [15]. The structured communication tool ICIT facilitates and organizes the utilization of CBT in primary care settings, where the presence of a structured session agenda may be crucial. In a previous study, we have documented the feasibility of implementing the structured communication tool ICIT in primary care settings in Norway [16].To enhance the validity of the structured communication tool ICIT, it incorporates a set of cognitive work tools that I have found to be practical and effective in primary care. These tools encompass: 1) problem-solving treatment, 2) behavioral activation, 3) Socratic dialogue, and 4) cognitive restructuring during the assessment of sick leave.

1: Problem-solving treatment

Problem-solving therapy (PST) serves as the cornerstone of the ICIT and is widely recognized as a well-established approach within the field of CBT [17]. Franke et al found problem-solving treatment feasible for the GPs to treat patients with emotional or psychosocial problems[18]. Based on experience in the United States, it is evident that GPs are inclined to continue utilizing PST [19]. PST is a concise and psychosocial intervention that equips patients with a systematic approach to effectively tackle everyday life challenges. The efficacy of this treatment in alleviating

symptoms of depression and other emotional distress has been well-documented in numerous studies[20,21]. Furthermore, PST has shown promising results in enhancing self-efficacy among individuals with various conditions, including diabetes, low-back pain, and osteoarthritis [22-24]. Empirical evidence supports its effectiveness in addressing various mental health concerns and facilitating adaptation in individuals dealing with physical conditions such as cancer, obesity, diabetes, and chronic pain [17,25]. The utilization of PST within the structured communication tool ICIT has the potential to enhance its efficacy as a transdiagnostic structured communication tool.

Patients visiting their GP may often experience a sense of being overwhelmed by various problems. Problem-solving therapy has been proven to be as effective as other treatments for mental disorders and adaptation to medical conditions. This therapeutic approach equips patients with step-by-step problem-solving skills, including: 1) identifying the problems, 2) categorizing the problems into actionable and non-actionable ones, 3) exploring alternative solutions and evaluating the most suitable options, 4) implementing the selected solutions, and 5) assessing the effectiveness of the problem-solving efforts [17].

How problem-solving treatment is used in the structured communication tool ICIT

The GP use the condensed version of the ICIT serving as a manual during encounters with general practitioners ,see figure 3. When implementing problem-solving treatment within the ICIT, the GPs collaborate with the patient to create a problem list, as illustrated in Figure 4.

3b.PROBLEM LIST

"To better assist you, I would like to get an overview of all the issues you feel are important, without delving into the details immediately. It's akin to a game of cards, where we need to distribute all the cards before we can start playing. Once we have a full picture, we can work together to address the issues at hand."

Review the points with the patient and ask:

"What are the problems that you can currently control, and which ones are beyond your control?"

Encourage the patient to select a problem that they believe is solvable. Write down step-by-step actions that can be taken to address this problem, including:

"What has helped in similar situations in the past?"
"Is there someone who can assist you with this?"

By using this approach, we can work collaborate to address the patient's concerns and develop a plan of action that is both realistic and effective.

Figure 4: The use of problem list in the structured communication tool ICIT

Within the structured communication tool ICIT, a streamlined adaptation of problem-solving therapy known as problem management is utilized. Problem-solving is a fundamental technique employed within the behavioral aspect of CBT. The primary objective is to assist patients in identifying their problems, which are subsequently documented as concise bullet points in the patients' medical record. Subsequently, the patient is encouraged to engage in problem sorting, distinguishing between issues that are currently unsolvable and those that can be addressed. In certain cases, the treatment approach involves accepting problems that are beyond the patient's control and cannot be solved. The patient is then prompted to prioritize the problems amenable to resolution by developing an activity plan, which is meticulously documented by their GP in the patient's medical record, using the patient's own expressions. The goal of the GP is to enhance patient problem-solving abilities through the utilization of open Socratic questions outlined in the ICIT manual. To facilitate this process, a step-by-step problem-solving approach is employed, and the details are diligently documented in the journal under the corresponding problem. This record is then printed out and assigned as homework for the upcoming session.

2: Behavioral activation

Peter Lewisohn's behavioral theory of depression[26] emerged in the 1970s, coinciding with Aaron T. Beck's cognitive therapy. Individuals experiencing depression commonly experience feelings of helplessness and tend to withdraw socially. Moreover, patients with MUPS often exhibit higher rates of depression and anxiety [27]. Behavioral activation, a therapeutic approach, involves actively motivating patients to partake in activities that foster a sense of achievement and happiness in their lives. This can involve contributing to their families, work, and the community [15]. Behavioral activation encompasses two essential techniques: 1) behavioral monitoring, where patients maintain a record of their activities in between sessions, and 2) activity planning, where patients devise plans centered around mastery and enjoyment. Remarkably, behavioral activation has been recognized as an equally effective treatment for depression when compared to full CBT [28].

Sometimes, patients may experience a sense of low self-efficacy in managing their personal or professional lives. Research suggests that patients should receive assistance in identifying effective coping strategies, while GPs should work towards fostering positive treatment expectations [29]. Behavior change may precede changes in beliefs [30].One effective approach could involve guiding patients towards behavioral changes to positively impact their beliefs.

<u>How behavioral activation is used in the structured communication tool ICIT</u>

When GPs utilize the structured communication tool ICIT, the primary focus is on the action phase of the consultation, which concludes with an activity plan. This plan involves initiating behavior changes either within the patient's work context, referred to as the "job list," or in their daily life through the "list of opportunity" or "problem list". In cases where a patient expresses low energy and diminished self-confidence, the ICIT recommends utilizing the "list of opportunity." Within this framework, GPs employ a predetermined set of Socratic questions to delve into how the patient's symptoms impact their everyday life, as suggested in figure 5. The objective is to collaboratively develop an activity plan within the patient's medical records, concentrating on achievable goals as perceived by the patient.

For instance, one question may involve assisting the patient in planning and organizing periods of rest to regain sufficient energy, enabling them to participate in social activities they had previously withdrawn from due to their health condition.

3c. LIST OF OPPORTUNITY

"A list of opportunity" can be helpful for patients who are struggling with a lack of motivation, feelings of helplessness, and social isolation. Some useful prompts for this list include:

- *"I'd like to understand how these problems are affecting your life. Is there anything that typically brings you joy that you find difficult to do now?"*
- *"Are there any activities that you usually enjoy that you still find fulfilling?"*
- *"What activities do you find restful and revitalizing? And is it beneficial to have a structured approach to resting, including when and how you rest?"*
- *"In what ways can your partner, friends, or family provide support that would be helpful given your current situation?"*

Using this "list of opportunity «can help patients identify opportunities for positive change and develop a sense of control in their lives."
To help patients prioritize and plan their next steps, create a bullet-pointed list of potential actions, and ask:

'Which of these actions do you feel would be the easiest for you to start with?'
Once a particular action has been identified, create a bullet-pointed plan for how it can be carried out. Then, ask the patient:

'How can we make this plan more feasible for you?'

By involving the patient in the process of identifying and planning actions that feel manageable and realistic, we can help them build a sense of competence and momentum towards positive change."

Figure 5: The use of "list of opportunity" in the structured communication tool ICIT

This process also allows the GP to gain a deeper understanding of the impact of the patient's health issues on their life. The GP´s objective is to shift the focus from challenges to opportunities, which are recorded as bullet points in the journal under the "list of opportunity." Once all the points have been identified, the GP asks the patient, "Which one would be the easiest for you to start with?" Based on the patient's choice, a concrete activity plan is developed. The goal is to empower the patient. Therefore, it is the GP´s responsibility to assist the patient in creating a detailed, realistic, and achievable plan as homework for the next session, which is printed out for reference. The agenda for

the subsequent session involves reviewing the activity plan and evaluating whether the chosen activity has brought a sense of mastery and joy to the patient.

3: Socratic dialogue

Socratic dialogue plays a central role in cognitive-behavioral therapy (CBT) by encouraging patients to examine the validity and helpfulness of their thoughts. Socratic questions are especially valuable in cognitive restructuring but are employed in various cognitive techniques. In cognitive therapy, challenging patients to reflect on the validity and usefulness of their own perceptions and thoughts is an integral part of the process. The use of friendly and inquisitive questions helps establish a therapeutic alliance between the therapist and the patient, which is crucial for effective therapy [31]. While Socratic questioning is integrated into CBT, its specific impact has been minimally studied. However, a study discovered a correlation between therapists employing Socratic questions and symptom improvement in cognitive therapy for depression [32].

How Socratic dialogue is used in the structured communication tool ICIT

The structured communication tool ICIT encompasses a predefined set of Socratic questions, which are open-ended in nature and designed to facilitate patient reflection on challenges they may be facing in their personal or professional life. Notably, all the questions within the ICIT framework adopt a salutogenic approach, emphasizing factors that promote health and well-being.

4: Cognitive restructuring

Cognitive restructuring involves a therapeutic process where the therapist assists patients in recognizing, evaluating, and, if necessary, modifying negative thoughts. In CBT, cognitive restructuring is applied to thoughts that arise in specific situations during times of stress or adversity, commonly referred to as automatic negative thoughts, as well as negative underlying beliefs[15].

Let's consider an example where a patient expresses the thought, "My boss doesn't like me, and I need to take a sick leave." While this thought may hold some truth, the GP's role is to help the patient examine the evidence supporting this belief. For instance, the GP may ask the following questions: 1)"Have you received any specific feedback from your boss?" or 2) "How did your most recent performance evaluation or appraisal go?"

By engaging in Socratic dialogue, the GP can assist the patient in uncovering automatic negative thoughts, while exploring their validity and promoting the development of more realistic and helpful thinking patterns. The existing literature highlights the importance of investigating the effectiveness of cognitive restructuring as a standalone intervention, as there is limited research available on this specific aspect[15].

How cognitive restructuring is used in the structured communication tool ICIT

In the structured communication tool ICIT, the cognitive restructuring treatment strategy is employed when discussing sick leave during consultations, among other approaches. Sick leave is regarded as a form of treatment with both its effects and side effects. The GP engages in Socratic guidance to examine the validity of the belief "I cannot work" and to explore whether it is genuinely impossible for the patient to be present at work.

By posing the question, "What would be beneficial for me at work given my current situation?" the patient is encouraged to reflect on possibilities and consider what they can feasibly accomplish at work despite their health issues. The GP records the patient's thoughts about what would be advantageous for them in the workplace directly in the journal, creating an activity plan known as the "job list", as shown in figure 6. Like the "possibility list," it is the therapist's responsibility to guide the patient in ensuring that the plan is realistic and attainable. If the GP determines that sick leave, whether full-time or part-time, is the most suitable treatment option for the patient, a copy of the "job list" is included in the sick leave note. This instructs the employer to make necessary adjustments to support the patient's ongoing employment. Moreover, it provides the Norwegian Labor and Welfare Agency (NAV) with a comprehensive assessment of the patient's work participation, focusing on their abilities at work rather than solely considering their limitations.

3a. JOB LIST

Taking sick leave =treatment.

However, it's worth noticing that being at work may have positive effects on your health.

- Considering asking the patient exploratory questions to help them identify positive aspects of work and areas that could be improved, such as:

• *"What aspects of work would be helpful for you given your current situation?"*

•*" Can you identify something positive about your work despite your symptoms?"*

• *"Are there specific tasks or aspects of work that bring you joy or satisfaction?"*

•*" Are there any tasks that are particularly challenging right now, and that you would benefit from delegating or seeking support for?"*

- Create a specific and detailed list in the patients 'medical record outlining the accommodations that could assist them in managing their symptoms while at work.
- This job list can serve as a basis for discussing potential adjustments or facilitate on with their employer.

Figure 6: The use of the "job list" in the structured communication tool ICIT

What is new about the structured communication tool ICIT?

- The structured communication tool ICIT is a condensed version of CBT, specifically designed for, and found feasible in primary care [16] . The ICIT offers a menu of simplified cognitive tools, including: 1) problem-solving treatment, 2) behavioral activation, 3) Socratic dialogue, and 4) cognitive restructuring during the assessment of sick leave.
- The structured communication tool ICIT is tangible, with written documentation that GPs can easily access as a manual during consultations.
- GPs consciously and systematically apply cognitive techniques in a structured manner, complete with documentation and assigning homework for unscheduled general practice visits.
- GPs employ the "MUPS explanatory model" to foster a shared understanding of patients' ailments, enabling physicians to move beyond the surface level of symptoms and delve deeper into the underlying factors. This approach facilitates a comprehensive comprehension of the patients' condition, considering various physical, psychological, and social factors that may contribute to their symptoms. By employing the MUPS explanatory model, GPs aim to gain a holistic understanding of the patient's health, which in turn enhances the quality of care provided and supports more targeted and effective treatment strategies.
- The cognitive techniques employed are simplified yet effective, empowering physicians to enhance their proficiency in working with patients.
- The structured communication tool ICIT includes a sick leave tool where cognitive restructuring assists GPs in exploring whether sick leave is a beneficial treatment option for patients.
- GPs undergo specialized training to adeptly recognize, plan, and implement cognitive techniques in the treatment of patients with MUPS, all while trying to ensure that patients do not experience any sense of stigmatization.

Reference list

1. Henningsen P, Zipfel S, Sattel H, Creed F. Management of functional somatic syndromes and bodily distress. *Psychotherapy and Psychosomatics* 2018; **87**(1): 12-31.
2. Chalder T, Willis C. “Lumping” and “splitting” medically unexplained symptoms: is there a role for a transdiagnostic approach? : Taylor & Francis; 2017. p. 187-91.
3. Wortman MS, van der Wouden JC, Twisk JW, et al. Effectiveness of psychosomatic therapy for patients with persistent somatic symptoms: Results from the CORPUS randomised controlled trial in primary care. *Journal of Psychosomatic Research* 2023: 111178.
4. Jadhakhan F, Romeu D, Lindner O, Blakemore A, Guthrie E. Prevalence of medically unexplained symptoms in adults who are high users of healthcare services and magnitude of associated costs: a systematic review. *BMJ Open* 2022; **12**(10): e059971.
5. Rask MT, Rosendal M, Fenger-Grøn M, Bro F, Ørnbøl E, Fink P. Sick leave and work disability in primary care patients with recent-onset multiple medically unexplained symptoms and persistent somatoform disorders: a 10-year follow-up of the FIP study. *General hospital psychiatry* 2015; **37**(1): 53-9.
6. Smith RC, Lein C, Collins C, et al. Treating patients with medically unexplained symptoms in primary care. *Journal of general internal medicine* 2003; **18**(6): 478-89.
7. Olde Hartman TC, Rosendal M, Aamland A, et al.
. *BJGP Open* 2017; **1**(3): bjgpopen17X101061.
8. olde Hartman TC, Rosendal M, Aamland A, et al. What do guidelines and systematic reviews tell us about the management of medically unexplained symptoms in primary care? *BJGP open* 2017; **1**(3).
9. Barsky AJ, Borus JF. Functional somatic syndromes. *Annals of internal medicine* 1999; **130**(11): 910-21.
10. Ursin H, Eriksen HR. Cognitive activation theory of stress (CATS). *Neuroscience & Biobehavioral Reviews* 2010; **34**(6): 877-81.
11. McEwen BS. Protective and damaging effects of stress mediators. *New England journal of medicine* 1998; **338**(3): 171-9.
12. Juster R-P, McEwen BS, Lupien SJ. Allostatic load biomarkers of chronic stress and impact on health and cognition. *Neuroscience & Biobehavioral Reviews* 2010; **35**(1): 2-16.
13. McEwen BS, Gianaros PJ. Central role of the brain in stress and adaptation: links to socioeconomic status, health, and disease. *Annals of the New York Academy of Sciences* 2010; **1186**(1): 190-222.
14. McEwen BS, Wingfield JC. The concept of allostasis in biology and biomedicine. *Hormones and behavior* 2003; **43**(1): 2-15.
15. Wenzel A. Basic strategies of cognitive behavioral therapy. *Psychiatric Clinics* 2017; **40**(4): 597-609.
16. Abrahamsen C, Lindbaek M, Werner EL. Experiences with a structured conversation tool: a qualitative study on feasibility in general practice in Norway. *Scand J Prim Health Care* 2022: 1-7.
17. D’Zurilla TJ, Nezu AM. Problem-solving therapy. *Handbook of cognitive-behavioral therapies* 2010; **3**: 197-225.
18. Franke LJ, van Weel-Baumgarten EM, Lucassen PL, Beek MM, Mynors-Wallis L, van Weel C. Feasibility of training in problem-solving treatment for general practice registrars. *European Journal of General Practice* 2007; **13**(4): 243-5.
19. Hegel MT, Dietrich AJ, Seville JL, Jordan CB. Training residents in problem-solving treatment of depression: a pilot feasibility and impact study. *Family medicine* 2004; **36**(3): 204-8.
20. Mynors-Wallis LM, Gath D, Lloyd-Thomas A, Tomlinson D. Randomised controlled trial comparing problem solving treatment with amitriptyline and placebo for major depression in primary care. *Bmj* 1995; **310**(6977): 441-5.
21. Catalan J, Gath D, Anastasiades P, Bond S, Day A, Hall L. Evaluation of a brief psychological treatment for emotional disorders in primary care. *Psychological medicine* 1991; **21**(4): 1013-8.
22. van den Hout JH, Vlaeyen JW, Heuts PH, Zijlema JH, Wijnen JA. Secondary prevention of work-related disability in nonspecific low back pain: does problem-solving therapy help? A randomized clinical trial. *The Clinical journal of pain* 2003; **19**(2): 87-96.
23. Didjurgeit U, Kruse J, Schmitz N, Stückenschneider P, Sawicki P. A time-limited, problem-orientated psychotherapeutic intervention in Type 1 diabetic patients with complications: a randomized controlled trial. *Diabetic Medicine* 2002; **19**(10): 814-21.
24. Heuts PH, De Bie R, Drietelaar M, et al. Self-management in osteoarthritis of hip or knee: a randomized clinical trial in a primary healthcare setting. *The Journal of Rheumatology* 2005; **32**(3): 543-9.
25. Nezu AM, Nezu CM, D'Zurilla T. Problem-solving therapy: A treatment manual: springer publishing company; 2012.
26. Lewinsohn PM. A behavioral approach to depression. *Essential papers on depression* 1974: 150-72.
27. Toft T, Fink P, Oernboel E, Christensen K, Frostholm L, Olesen F. Mental disorders in primary care: prevalence and co-morbidity among disorders. Results from the functional illness in primary care (FIP) study. *Psychological medicine* 2005; **35**(8): 1175-84.

28. Dimidjian S, Hollon SD, Dobson KS, et al. Randomized trial of behavioral activation, cognitive therapy, and antidepressant medication in the acute treatment of adults with major depression. *Journal of consulting and clinical psychology* 2006; **74**(4): 658.
29. Pourová M, Klocek A, Řiháček T, Čevelíček M. Therapeutic change mechanisms in adults with medically unexplained physical symptoms: A systematic review. *Journal of psychosomatic research* 2020; **134**: 110124.
30. Reme S, Stahl D, Kennedy T, Jones R, Darnley S, Chalder T. Mediators of change in cognitive behaviour therapy and mebeverine for irritable bowel syndrome. *Psychological medicine* 2011; **41**(12): 2669-79.
31. Heins MJ, Knoop H, Bleijenberg G. The role of the therapeutic relationship in cognitive behaviour therapy for chronic fatigue syndrome. *Behaviour research and therapy* 2013; **51**(7): 368-76.
32. Braun JD, Strunk DR, Sasso KE, Cooper AA. Therapist use of Socratic questioning predicts session-to-session symptom change in cognitive therapy for depression. *Behaviour research and therapy* 2015; **70**: 32-7.

Supplementary material 2

ICIT complete version

Individual Challenge Inventory Tool (ICIT) is a structured communication tool with a work-focused approach based on cognitive therapy

1.SET THE AGENDA FOR THE SESSION
Greet and introduce yourself.
Apologize, if necessary, for example, if you are late.
Engage in small talk until the patient is seated.
Agenda setting: "*What do we need to talk about today*?" and "*What else?"*

2.VALIDATION - Confirm the patient's feelings
•Validate the patient's feelings with praise: *"I'm glad you came today. It's clear that you don't want to feel this way."*
•Acknowledge the patient's situation: *"It's understandable that you feel tired given your condition. It's not how you would like things to be."*
•Legitimize the patient's experience: *"It's completely normal to feel fatigued and exhausted when you've been experiencing pain and discomfort for a prolonged period."*
•Confirm the patient's feelings: *"I understand that coping with your pain/headache/digestive problems can be difficult, and I would like to gain a better understanding of how they are impacting your daily life."*
•For patients with MUPS: *"Research indicates that persistent stress can have a significant impact on your body, contributing to the symptoms you are experiencing."*
•Provide support and insight: *"I would like to offer you a method that may assist with managing your symptoms. Would you be open to trying it together?"*

Step-by-step approach by patients with medically unexplained physical symptoms:

"To help you with your health concerns, we'll be using a step-by-step approach. Firstly, we'll summarize what we have discussed thus far, then we'll collaborate to find a possible explanation for your symptoms, and lastly, we'll develop a plan to help improve your wellbeing. To achieve this effectively, we'll require scheduling three appointments soon. Does this plan work for you?"

3a: CONSULTATION NUMBER 1
"During our first consultation, let's review the tests and findings we've gathered so far."

If possible, try to explain for the patient their condition whether it's non-cardiac chest pain, fibromyalgia, tension headaches, tinnitus, or something else.

The doctor may say:

- *"It's important to recognize that the body and mind are connected, especially in cases like this. For instance, our heart rate increases when we feel afraid, and it slows down once the perceived danger has passed. This is normal.*
- *"I want you to know that I believe you, and it's not uncommon for doctors to be unable to determine the underlying cause of health issues through blood tests or other medical exams. For instance, after an infection, it's typical to feel exhausted and sluggish, even after the infection has resolved."*
- *"Fortunately, ongoing research is exploring how we can provide effective treatments for patients in your condition. There is help available, and it is possible for you to improve."*

- *"Lastly, do you have any ideas or thoughts on what might be causing your symptoms?*
- *"Are there any patterns or connections you've noticed? Let's discuss this further during our next session."*

3b: CONSULTATION NUMBER 2

- Use the MUPS explanatory model to create a shared understanding of the symptoms:

"We've learned a lot about what might be causing your symptoms, and I have a visual model that can help us understand this together. Would you be okay with me showing it to you?"

- Provide the patient with their own copy of the model in a printed handout.
- "What triggers long-term stress in your life?" Identifying maintaining factors is a part of the treatment process. Ask the patient to complete either the "problem list" or the "list of opportunity " on the MUPS explanatory model, which we'll discuss in our next session.
- It's important not to jump into implementing solutions during consultation no. 2. The patient needs time to digest the MUPS explanatory model, and it's essential for them to reflect on what they believe is sustaining long-term stress in their life. By refraining from implementing solutions at this stage, we can give the patient the space and time they need to fully engage with the process of understanding their symptoms and identifying potential solutions.

3c: CONSULTATION NUMBER 3: ICIT

Consultation No. 3 is the ICIT action phase of the consultation. It's important to start this phase promptly to utilize the time effectively. The following is a list of interventions that the doctor can use based on the patient's situation. The aim is to develop a short-written plan using either «*list of opportunity,", "problem list"* or *"job list",* which may be combined depending on the patient's needs.

4A. "LIST OF OPPORTUNITY"

"A list of opportunity" can be helpful for patients who are struggling with a lack of motivation, feelings of helplessness, and social isolation. Some useful prompts for this list include:

- *"I'd like to understand how these problems are affecting your life. Is there anything that typically brings you joy that you find difficult to do now?"*
- *"Are there any activities that you usually enjoy that you still find fulfilling?"*
- *"What activities do you find restful and revitalizing? And is it beneficial to have a structured approach to resting, including when and how you rest?"*
- *"In what ways can your partner, friends, or family provide support that would be helpful given your current situation?"*

Using the "*list of opportunities*" can help patients identify opportunities for positive change and develop a sense of control in their lives. During the consultation, ask the patient very specifically about what they like to do that can reduce their symptoms. Getting help with planning rest, such as energy conservation, can be helpful. Discuss how the patient plans to structure their rest, what it takes to, for example, follow the child to football training, where the patient rests best, and for how long.
Some patients may need some time to respond, so don't be afraid of silence!
To help patients prioritize and plan their next steps of potential actions the doctor may ask:

"Which of these actions do you feel would be the easiest for you to start with?"

Once a particular action has been identified, create a bullet-pointed plan for how it can be carried out, and then ask the patient:

"How can we make this plan more feasible for you?"

Create a short, concrete, detailed, and feasible plan together with the patient based on points from section. By involving the patient in the process of identifying and planning actions that feel manageable and realistic, we can help them build a sense of competence and momentum towards positive change.
It is essential to ask the patient, "Is this feasible?" when creating an activity plan. The primary objective of the activity plan is to support the patient in coping better with their symptoms.

4B: "PROBLEM LIST"
"To better assist you, I would like to get an overview of all the issues you feel are important, without delving into the details immediately. It's akin to a game of cards, where we need to distribute all the cards before we can start playing. Once we have a full picture, we can work together to address the issues at hand."

Review the points with the patient and ask:
"What are the problems that you can currently control, and which ones are beyond your control?"

Encourage the patient to select a problem that they believe is solvable. Write down step-by-step actions that can be taken to address this problem, including:

"What has helped in similar situations in the past?"
"Is there someone who can assist you with this?"

By using this approach, we can work collaboratively to address the patient's concerns and develop a plan of action that is both realistic and feasible.

4C: "JOB LIST"
Sick leave can serve as a treatment option, but it may not always be the best course of action. In some cases, taking sick leave can validate avoidance behavior that could ultimately worsen the individual's condition. Research indicates that being at work generally has positive effects on an individual's health. Therefore, it may be helpful for the doctor to challenge patients' beliefs that "*working will make me sicker."* By asking targeted questions, doctors can explore the validity of such beliefs and work to modify them.
Having a conversation tool for sick leave assessment can be useful in shifting the patient's focus from problems to opportunities. As an introduction to this evaluation, doctors can present a "job list" that includes five questions to help gain a better understanding of the patient's job. To ensure accuracy, the doctor can ask permission to write down their responses while discussing the list, saying something like:

"I don't know much about your job, so I have a few questions on this 'job list' that could help us. Would it be alright if I write down your answers as we go along?"

• *"What aspects of work would be helpful for you given your current situation?"*
• *" Can you identify something positive about your work despite your symptoms?"*
• *"Are there specific tasks or aspects of work that bring you joy or satisfaction?"*
• *" Are there any tasks that are particularly challenging right now, and that you would benefit from delegating or seeking support for?"*

Write it down in the journal point by point and, send it in writing with the patient. The list can be the starting point for a job conversation with the manager where adjustments are needed.

5.FOLLOW-UP in a new consultation within 2 weeks.
Review the homework. It is therefore crucial to record the information in the journal. Homework refers to carrying out the activity plan that has been created, whether it be in the form of a "*list of opportunity*"," "*problem list,"* or "*job list*." In cognitive therapy, homework is considered an essential component. It is often not completed if it is not written down and printed out. To conclude the consultation, the doctor can adopt the role of a coach and encourage the patient to think of the activity plan as a training plan that they will try. While the doctor cannot complete the homework for the patient, they can express their interest in learning about the patient's progress during the next appointment. The doctor could conclude the session by saying something like:

"I'll be your coach in this process. We've created a training plan that you'll try. I can't do the training for you, but I'm eager to hear about your progress at our next appointment."

Supplementary material 3

Study protocol

Study Design

This study adopts a cluster randomized controlled trial (cRCT) design, with randomization of General Practitioners (GPs).

Objectives

To compare the effectiveness of a structured communication tool, the Individual Challenge Inventory Tool (ICIT), based on work-focused cognitive-behavioral therapy with usual care in patients with medically unexplained physical symptoms (MUPS).

Materials

In October 2021, the Norwegian Medical Association approved a clinical communication course to train the GPs in using the structured communication tool ICIT. A total of 106 GPs were randomly assigned to attend the course on either March 4-5, 2022 (intervention group) or May 20-21, 2022 (usual care group).

Primary Outcome Measure

The Patient Global Impression of Change (PGIC) was selected as the primary outcome measure for this study. The PGIC assesses changes in clinical status based on patient-reported experiences of changes in function, symptoms, and quality of life from baseline to follow-up. It evaluates overall changes in function, symptoms, and quality of life perceived by participants, and involves asking one question: *"Describe the changes in function, symptoms, and overall quality of life since you received treatment by your GP."* Participants are then provided with seven response alternatives ranging from "very much better" to "very much worse. «PGIC has been validated and is commonly used as an outcome measure when objective measures or biomarkers are not feasible. It represents a clinically relevant tool for evaluating the perceived effectiveness of disease treatment [1].

Secondary outcome measures

1.The Patient Experience Questionnaire (PEQ) is completed by patients after their last session in the study with the GP in the usual care and intervention groups. The PEQ, developed for primary health care, assesses patient interactions, emotions, and counseling outcomes [2].

2.Health-related quality of life (HRQoL): We used the RAND-36, a 36-item measure evaluating eight aspects of health, including physical functioning, role limitations caused by physical and emotional problems, social functioning, emotional well-being, energy/fatigue, pain, and general health perceptions [3]. We aim to assess changes in patients' symptoms when GPs use the communication tool ICIT compared to usual care. We will also examine changes in patients' symptoms before and after treatment with ICIT. The questionnaire has been translated into Norwegian as RAND-36 “Your Health”.

3.Possible depression: Patients are asked, *"Have you felt sad or depressed in the last 14 days?"* Studies have validated a single question to assess possible depression in patients, and we aim to explore how any depression occurrence affects the patient's ability to benefit from ICIT [4].

4.Self-efficacy return-to-work questionnaire (RTW-SE): We used a validated 11-item questionnaire to assess work-related self-efficacy in individuals with common mental disorders [5]. Scores of 4.6 and above indicate a high probability of full-time employment without sick leave, while scores from 3.7 and below indicates a high probability of full sick leave [6]. We aim to investigate whether the GPs' use of ICIT can increase patients' selfefficacy for returning to work.

4.Sick leave: The GPs recorded sick leave at baseline, after the last session in the study, and at 11 weeks.

5.GPs' self-assessment of their ability to manage sick leave evaluations for patients with MUPS: We used a customized version of the PGIC in a questionnaire to all doctors in the study. We aim to investigate whether the GPs create a written concrete plan or discuss what it takes for patients to be at work despite their symptoms.

6.Patients' attitude toward creating a concrete plan with their GP for what they can do at work despite their symptoms: We used a customized version of the PGIC to measure this.

7.We aim to examine any change in the doctor's ability to manage sick leave evaluations after learning and using ICIT in their practice using a customized version of the PGIC.

The Intervention

General Practitioners (GPs) participated in a 25-hour training program which involved a two-day course comprising of lectures and practical training with up to 10 patients using the ICIT communication tool. To aid in the use of the communication tool ICIT, GPs received a laminated, condensed version of the tool for use during the course and with their patients. The training was based on Bandura's social learning theory and consisted of four stages: (i) attention, (ii) theoretical training, (iii) role-playing challenging scenarios, and (iv) motivation to imitate observed behavior (Bandura, 1977). The GPs were demonstrated the individual elements of the ICIT via video consultations and were then organized into groups to role-play cases from their own practice in triads, taking turns to play the roles of doctor, patient, and observer. This educational method is commonly used in guidance groups as part of the training for general practitioners. As part of the course, the GPs were invited to attend digital follow-up meetings in groups for guidance and reinforcement of knowledge during the 11-week study period.

Randomization

Randomization of doctors into the intervention and usual care groups was carried out at the group level on January 17, 2022, at the University of Oslo (UIO).

Selection of GPs

The recruitment of general practitioners (GPs) was conducted by means of enrollment in the course, which was made available through the Medical Association's course catalog to all eligible GPs in the country. The eligibility criteria required that the participants be currently practicing as GPs and possess a high level of proficiency in the Norwegian language.

GP variables

The study collected data on several variables related to participating GPs, including their gender, age, years of experience in general practice, whether they work in solo or group practice, whether they are specialists or not specialists, whether they work in urban or rural areas, and any previous training they have received, including cognitive therapy courses.

Participant variables

The study collected data on several variables related to the participating individuals, including their gender, age, marital status, number of children, and education level.

Selection of participants

The study recruited participants based on the following set of criteria:

Inclusion Criteria

- All patients with medically unexplained physical symptoms (MUPS), such as persistent pain, headaches, dizziness, fatigue, irritable bowel syndrome, and musculoskeletal symptoms, were included in the study.
- The patients had to experience functional impairment, which was manifested as either sick leave (continuous or intermittent) or withdrawal from social and leisure activities due to MUPS.
- The patients were considered to have undergone sufficient medical investigations without any conclusive findings to explain their symptoms, and no further investigations were planned.
- The patients had experienced MUPS for at least three months continuously and were aged 18 years or over.

Exclusion Criteria

- Insufficient proficiency in the Norwegian language to complete the ICIT and/or respond to questionnaires.
- Continued medical investigation that MAY account for present symptoms and complaints.
- The patient disagrees with the doctor's evaluation of symptoms and complaints and desires further medical investigation.
- The patient declines to take part in the study.

• The patient must not have a dependence on alcohol or drugs.

What should the GPs register?

• We will assess the degree of occupational participation, which refers to the frequency and duration of sick leave or long-term benefits (> 12 months), at three time points: study start, after the last consultation, and at the end of the 11-week intervention period.
• A comprehensive list of all diagnoses that the patients have received in the last two years.
• A comprehensive list of all symptoms that the GPs identified as MUPS during the study period.

Project implementation

The study enrolled general practitioners who registered for a course and were randomly assigned to either the control or intervention group. The intervention group received the ICIT course on March 4-5, while the control group received it on May 20-21. All GPs agreed to participate in the study upon registering for the course and received a discounted fee as an incentive. Written consent was obtained from all doctors at the start of the study in March 2022.

Patient recruitment began in March, following the completion of the course for the intervention group. All doctors received the same letter regarding patient inclusion, and every Friday, they reviewed their patient lists for the following week to identify eligible participants based on the inclusion and exclusion criteria to avoid selection bias. Over an 11-week period, doctors in each group were required to include up to 10 patients.

The GPs contacted the patients and requested their participation. The patients received the information and consent forms in advance and were asked to arrive 20 minutes before their appointment to complete the forms and questionnaire. The consent and questionnaire forms were sealed separately in new envelopes, which the patients delivered to the reception. The GPs were provided with a form to record the patient's name, patient number, and whether they had submitted the forms before and after the consultation.

Patients in the control group received one or more consultations for their problem, while patients in the intervention group had at least two consultations due to the review of homework assignments at the follow-up appointment as part of the communication tool ICIT. All participants filled out the questionnaire again within 14 days after the last session in the study.

The GPs in the intervention group were required to print out the journal note in the form of "problem list," "possibility list," or "job list" for their 10 included patients, with a copy for the patient and an anonymized copy for the study. All doctors were blinded to the questionnaires.

Before the study began, all doctors signed a consent form and answered a questionnaire. They also answered a questionnaire after completing the ICIT course and using it on their patients. The degree of occupational participation (sick leave or disability benefits) was recorded for each patient at the first consultation, after the last

consultation, and after 11 weeks. The doctors also registered the patients' symptoms of MUPS, and all diagnoses the patients had in the past two years.

All doctors received written information about the logistics of the study and how to select patients. To ensure that the doctors in the control group were familiar with the inclusion and exclusion criteria, they were invited to a digital information meeting before the start of the study.

Power calculation

The power calculation was conducted using a 7-point scale that ranged from "very much better" (1) to "very much worse" (7). Despite the absence of prior studies examining the use of PGIC to evaluate MUPS patients, a previous investigation on chronic pain patients and their PGIC scores at 12 months was employed [7]. The average score on the 7-point scale was 3.5, with 1 indicating "very much better," 4 indicating no change, and 7 indicating "very much worse." To determine a clinically significant difference, we established a 20% change, which equated to a value of 4.2 before the intervention. Furthermore, we assumed that the control group's score would remain constant before and after the intervention, while the intervention group's score would shift from 4.2 to 3.5.

Moreover, we have taken into consideration an intra-cluster correlation coefficient (ICC) of 0.10, a statistical power of 90%, and a standard deviation of 1.36. Using these parameters, we have determined that a minimum of 70 doctors is required to recruit at least 3 patients each in both the control and intervention groups, yielding a total of 210 patients. We have also accounted for the possibility that some physicians may not be able to recruit the full quota of 10 patients, and that some patients may not complete the study.

Statistics

The statistical analysis of the data will involve the use of appropriate statistical tests for different types of variables. T-tests will be utilized for continuous variables, while chi-square tests will be used for proportions. Additionally, regression analysis will be employed to identify potential predictors of change.

Ethics

The study has been approved by REK (case number 387480) and is also seeking approval from NSD. The study is registered in Clinical Trials (NCT05128019) and will also be registered in Forskpro, at UiO.

Funding

The study was funded by The Norwegian Research Fund for General Practice.

Reference list

1. Rampakakis E, Ste-Marie PA, Sampalis JS, Karellis A, Shir Y, Fitzcharles M-A. Real-life assessment of the validity of paLent global impression of change in fibromyalgia. *RMD open* 2015; **1**(1): e000146.
2. Steine S, Finset A, Laerum E. A new, brief quesLonnaire (PEQ) developed in primary health care for measuring patients' experience of interaction, emotion, and consultation outcome. *Family prac1ce* 2001; **18**(4): 410-8.
3. Framework IC. The MOS 36-item short-form health survey (SF-36). *Med Care* 1992; **30**(6): 473-83.
4. Reme SE, Eriksen HR. Is one question enough to screen for depression? *Scandinavian journal of public health* 2010; **38**(6): 618-24.
5. Lagerveld SE, Blonk RW, Brenninkmeijer V, Schaufeli WB. Return to work among employees with mental health problems: development and validation of a self-efficacy questionnaire. *Work & Stress* 2010; **24**(4): 359-75.
6. Gjengedal RG, Lagerveld SE, Reme SE, Osnes K, Sandin K, Hjemdal O. The Return-to-Work Self-efficacy Questionnaire (RTW-SE): A Validation Study of Predicative Abilities and Cut off Values for Patients on Sick Leave Due to Anxiety or Depression. *Journal of Occupa1onal Rehabilita1on* 2021: 1-10.
7. Reme SE, Ljosaa TM, Stubhaug A, Granan LP, Falk RS, Jacobsen HB. Perceived Injustice in Patients with Chronic Pain: Prevalence, Relevance, and Associations with Long-Term Recovery and Deterioration. *J Pain* 2022; **23**(7): 1196-207.

Supplementary material 4

The mixed model linear regressions were estimated Stata 17 using code with this structure:

```
mixed y x1 x2 || GPid || PASid
        estat icc
```

Here, y represents the dependent variable, while x1 and x2 represent two independent variables. The variables GPid and PASid denote the GP identity and the patient identity, respectively, and "|| GPid" and "|| Pasid" specify that the model should include random intercepts at two levels. The second line, estat icc, specifies that the residual intraclass correlation should be calculated.

The definitions of the intraclass correlations (ICCs) are based on the variance of the regression's error term, σ_ϵ^2, the variance of the patient intercepts, σ_{pas}^2, and the variance of the GP intercepts σ_{GP}^2. In table S1, the ICC for lines with level GP is defined as $\sigma_{GP}^2 / (\sigma_\epsilon^2 + \sigma_{GP}^2)$, while the ICC for lines with level Pas|GP is defined as $(\sigma_{GP}^2 + \sigma_{pas}^2) / (\sigma_\epsilon^2 + \sigma_{GP}^2 + \sigma_{pas}^2)$.

Table S1· Intraclass correlation to supplement to mixed model linear regressions reported in Table 3

		Unadjusted		Adjusted	
Dependent variables	Level	ICC	95% CI	ICC	95% CI
Patient Global impression of Change	GP	0·025	(0·002 to 0·253)	0·080	(0·020 to 0·266)
Work related self-efficacy	GP	0·019	(0·000 to 0·695)	0·000	(0·000 to 0·000)
	Pas|GP	0·703	(0·630 to 0·767)	0·663	(0·561 to 0·752)
Physical functioning	GP	0·105	(0·053 to 0·198)	0·030	(0·002 to 0·343)
	Pas|GP	0·836	(0·806 to 0·862)	0·788	(0·740 to 0·830)
	GP	0·028	(0·005 to 0·140)	0·025	(0·001 to 0·363)
Role limitations due to physical health	Pas|GP	0·511	(0·442 to 0·579)	0·443	(0·350 to 0·540)
	GP	0·000	(0·000 to 0·000)	0·000	(0·000 to 0·000)
Role limitations due to emotional problems	Pas|GP	0·538	(0·472 to 0·603)	0·496	(0·407 to 0·586)
Energy/fatigue	GP	0·010	(0·000 to 0·374)	0·000	(0·000 to 0·000)
	Pas|GP	0·610	(0·551 to 0·666)	0·575	(0·493 to 0·652)
Emotional well-being	GP	0·024	(0·004 to 0·142)	0·000	(0·000 to 0·000)
	Pas|GP	0·634	(0·577 to 0·688)	0·614	(0·537 to 0·686)
Social functioning	GP	0·045	(0·013 to 0·151)	0·000	(0·000 to 0·000)
	Pas|GP	0·687	(0·636 to 0·733)	0·655	(0·584 to 0·719)
Pain	GP	0·080	(0·035 to 0·172)	0·000	(0·000 to 0·000)
	Pas|GP	0·732	(0·687 to 0·774)	0·678	(0·609 to 0·739)
General health	GP	0·045	(0·013 to 0·143)	0·000	(0·000 to 0·000)
	Pas|GP	0·812	(0·780 to 0·841)	0·757	(0·703 to 0·803)
Communication	GP	0·126	(0·062 to 0·240)	0·165	(0·058 to 0·388)
Barriers	GP	0·043	(0·008 to 0·198)	0·067	(0·011 to 0·326)
Emotions	GP	0·014	(0·000 to 0·502)	0·046	(0·005 to 0·334)
Partial sick leave adjusted for full sick leave	GP	0·065	(0·016 to 0·228)	0·081	(0·015 to 0·329)
	Pas|GP	0·733	(0·678 to 0·781)	0·678	(0·596 to 0·750)

Intraclass correlation (ICC) with 95% confidence intervals (95% CI).
Outcomes observed once (at follow-up only) have random intercepts for physicians (GP), while outcomes observed twice (at baseline and follow-up) have random intercepts for patients in addition (Pas|GP). In cases where the ICC were zero, the regressions were estimated without the corresponding random intercept. This did not affect the results reported in Table 3.

Supplementary material 5

The structured communication tool ICIT - search strategies

Ovid MEDLINE(R) ALL <1946 to May 09, 2023>

1	medically unexplained symptoms/	938
2	Somatoform Disorders/	9592
3	(medically unexplained symptom* or mups or somatic symptom* or somatoform disorder* or conversation tool*).tw,kf.	10186
4	or/1-3	17354
5	General Practitioners/	10495
6	exp Primary Health Care/	190166
7	((general adj2 (practitioner* or physician*)) or (primary adj2 care)).tw,kf.	221997
8	or/5-7	341326
9	Sick Leave/	6722
10	((sick or medical or illness) adj2 (leave or days)).tw,kf.	8979
11	9 or 10	12663
12	4 and 8 and 11	34

Embase Classic+Embase <1947 to 2023 May 09>

1	medically unexplained symptom/	927
2	exp somatoform disorder/	32814
3	(medically unexplained symptom* or mups or somatic symptom* or somatoform disorder* or conversation tool*).tw,kf.	14891
4	1 or 3	15154
5	general practitioner/	122096
6	primary medical care/	131331
7	((general adj2 (practitioner* or physician*)) or (primary adj2 care)).tw,kf.	302675
8	or/5-7	376074
9	medical leave/	8750
10	((sick or medical or illness) adj2 (leave or days)).tw,kf.	12765
11	9 or 10	15968
12	4 and 8 and 11	22

APA PsycInfo <1806 to May Week 1 2023>

1	exp somatoform disorders/	14373
2	(medically unexplained symptom* or mups or somatic symptom* or somatoform disorder* or conversation tool*).mp.	18639
3	1 or 2	23957
4	general practitioners/	6342
5	primary health care/	21073
6	((general adj2 (practitioner* or physician*)) or (primary adj2 care)).mp.	54799
7	or/4-6	54799
8	employee leave benefits/	1479
9	((sick or medical or illness) adj2 (leave or days)).mp.	3007
10	8 or 9	3645
11	3 and 7 and 10	23

Cochrane Library
Date Run: 10/05/2023 03:11:46

ID Search Hits
#1 MeSH descriptor: [Medically Unexplained Symptoms] this term only 67
#2 MeSH descriptor: [Somatoform Disorders] explode all trees 887
#3 ("medically unexplained symptom*" OR mups OR "somatic symptom*" OR "somatoform disorder*" OR "conversation tool*"):ti,ab,kw 496
#4 {OR #1-#3} 1323
#5 MeSH descriptor: [General Practitioners] explode all trees 483
#6 MeSH descriptor: [Primary Health Care] explode all trees 10257
#7 ((general NEAR/2 (practitioner* OR physician*)) OR (primary NEAR/2 care)):ti,ab,kw 33020
#8 {OR #5-#7} 37074
#9 MeSH descriptor: [Sick Leave] this term only 657
#10 ((sick OR medical OR illness) NEAR/2 (leave OR days)):ti,ab,kw 2361
#11 #9 OR #10 2361
#12 #4 AND #8 AND #11 4

Supplementary material 6

General practitioner characteristics by treatment allocation

Table S 2. General practitioner characteristics by treatment allocation

Variable	Control (n=43)		Intervention (n=44)		Total (n=87)	
Age, mean (sd)	42.79	(9.81)	41.43	(8.81)	42.10	(9.29)
Female	26	(60.5%)	28	(63.6%)	54	(62.1%)
Specialist	22	(51.2%)	20	(45.5%)	42	(48.3%)
List length, mean (sd)[a)]	1024.39	(353.62)	960.70	(252.92)	991.79	(306.04)

The table entries are N (%) for categorical variables and mean (standard deviation) for numerical variables.

a) There were 2 and 1 missing list length values for the control and intervention groups, respectively.

The differences between the groups are not statistically significant.
